# The UBE2C/CDH1/DEPTOR axis is an oncogene and tumor suppressor cascade in lung cancer cells

**DOI:** 10.1172/JCI162434

**Published:** 2023-02-15

**Authors:** Shizhen Zhang, Xiahong You, Yawen Zheng, Yanwen Shen, Xiufang Xiong, Yi Sun

**Affiliations:** 1 Cancer Institute and; 2 Department of Breast Surgery and Oncology, Key Laboratory of Cancer Prevention and Intervention, Ministry of Education, the Second Affiliated Hospital, and; 3 Institute of Translational Medicine, Zhejiang University School of Medicine, Hangzhou, China.; 4 Zhejiang University Cancer Center, Hangzhou, China.; 5 Research Center for Life Science and Human Health, Binjiang Institute of Zhejiang University, Hangzhou, China.

**Keywords:** Cell Biology, Oncology, Lung cancer, Ubiquitin-proteosome system

## Abstract

Ubiquitin-conjugating enzyme E2C (UBE2C) mediates ubiquitylation chain formation via the K11 linkage. While previous in vitro studies showed that UBE2C plays a growth-promoting role in cancer cell lines, the underlying mechanism remains elusive. Still unknown is whether and how UBE2C plays a promoting role in vivo. Here we report that UBE2C was indeed essential for growth and survival of lung cancer cells harboring *Kras* mutations, and UBE2C was required for *Kras^G12D^*-induced lung tumorigenesis, since *Ube2c* deletion significantly inhibited tumor formation and extended the lifespan of mice. Mechanistically, *Kras^G12D^* induced expression of UBE2C, which coupled with APC/C^CDH1^ E3 ligase to promote ubiquitylation and degradation of DEPTOR, leading to activation of mTORC signaling. Importantly, DEPTOR levels fluctuated during cell cycle progression in a manner dependent on UBE2C and CDH1, indicating their physiological connection. Finally, *Deptor* deletion fully rescued the tumor inhibitory effect of *Ube2c* deletion in the *Kras^G12D^* lung tumor model, indicating a causal role of *Deptor*. Taken together, our study shows that the UBE2C/CDH1/DEPTOR axis forms an oncogene and tumor suppressor cascade that regulates cell cycle progression and autophagy and validates UBE2C an attractive target for lung cancer associated with *Kras* mutations.

## Graphical abstract

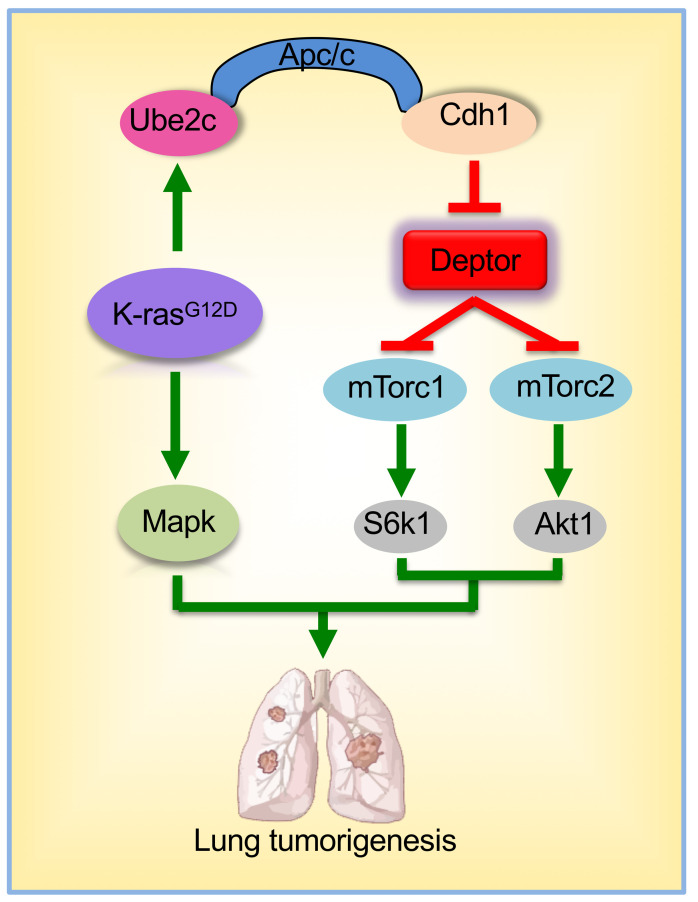

## Introduction

Protein ubiquitylation is a posttranslational modification that couples with the proteasome to form the ubiquitin-proteasome system (UPS). By controlling the ubiquitylation and degradation of more than 80% of normal and abnormal intracellular proteins, the UPS regulates diverse aspects of cell physiologic and pathologic processes (1). Biochemically, ubiquitylation is catalyzed step-wise by 3 types of enzymes: ubiquitin-activating enzymes (E1s), ubiquitin-conjugating enzymes (E2s), and ubiquitin ligases (E3s) (2). The family of E2s consists of approximately 40 members involved in the transfer of ubiquitin from E1s to E3s by governing ubiquitin chain initiation and elongation (3). Unlike common E2s such as CDC34 and UBEH5C that assemble the polyubiquitylation chain via the K48 linkage, ubiquitin-conjugating enzyme E2C (UBE2C, also known as UBCH10) and its partner E2 (UBE2S) initiate and extend, respectively, the ubiquitylation chain via the K11 linkage (4). It is well established that UBE2C/2S E2s couple with APC/C E3 ligase to promote ubiquitylation and degradation of cell cycle regulatory proteins (5). Interestingly, our recent studies showed that SAG-SCF E3 also works with E2s of UBE2C/2S for K11-linked ubiquitylation (6, 7), and it competes with APC/C for UBE2C/2S binding in a cell cycle–dependent manner to ensure the fidelity of cell cycle progression (8, 9).

UBE2C fluctuates with and is periodically activated during the cell cycle by initiating the ubiquitin modification of cell cycle–related proteins to ensure proper progression (10). A number of previous studies using various in vitro cell culture models have identified UBE2C as an oncogenic protein that is associated with malignant transformation (11–13). For example, ectopic *UBE2C* overexpression promoted, whereas *UBE2C* knockdown inhibited, cell proliferation in various cancer cell lines (14–16). Importantly, UBE2C is expressed at relatively low levels in many normal tissues, but at high levels in human carcinomas derived from the lung, uterus, bladder, and stomach (12), and high UBE2C expression is associated with worse overall survival of lung cancer patients (17–20). Generally, UBE2C exerts its oncogenic activities by promoting the polyubiquitylation and degradation of several cell cycle–related proteins (21, 22). Two cell culture–based studies also revealed that UBE2C could inhibit autophagy (16, 19). In a lung cancer cell model, UBE2C-mediated autophagy suppression was associated with growth, survival, and malignant phenotypes (19). However, the underlying mechanism by which UBE2C suppresses autophagy in lung cancer cells, and whether UBE2C plays an in vivo oncogenic role during lung tumorigenesis, are unknown.

The DEP domain–containing mechanistic target of rapamycin (mTOR) interacting protein (DEPTOR), also known as DEPDC6 (DEP domain–containing protein 6), serves as a negative regulator of both mTOR complex 1 (mTORC1) and complex 2 (mTORC2) (23). In in vitro cell culture studies, DEPTOR was initially characterized as a tumor suppressor, since its expression was downregulated in multiple tumors (24–32), and its depletion enhanced cell growth and survival by activating mTORC1 and mTORC2 signaling (24, 32, 33). On the other hand, DEPTOR could also activate AKT by relieving the feedback inhibition from mTORC1 to phosphoinositide 3-kinase (PI3K) signaling (23, 34, 35), or stabilize human epidermal growth factor receptor 2 (HER2) by abrogating β-TrCP–mediated degradation (36), or activate the TGF-β1/Smad3/Smad4/Snail pathway via mTOR inhibition (37). As such, DEPTOR also acts as an oncoprotein in certain cellular contexts (23, 36–40). Thus, it appears that DEPTOR has a rather complicated role in the regulation of growth and survival of cancer cells. However, 2 in vivo studies, using genetically modified *Deptor*-knockout (*Deptor*-KO) mouse models, showed that *Deptor* is a tumor suppressor, whose depletion (a) promoted lung tumorigenesis induced by the combination of *Kras^G12D^* activation and *p53* deletion via the activation of EGFR/mTORC signaling (26), and (b) accelerated prostate tumorigenesis triggered by *Pten* loss via the activation of mTORC signaling (24).

DEPTOR is known to be ubiquitylated and degraded by SCF^β-TrCP^ E3 ligase upon phosphorylation by S6K/RSK in response to mitogen stimulation (41–43). Given that DEPTOR plays an important role in regulation of various biological processes, including proliferation, autophagy, metabolism, and immunity (34, 44), it is very likely that DEPTOR levels could be regulated by additional E3 ligase(s). Currently, it is completely unknown which E3 under what physiological and pathological conditions will control the protein levels of DEPTOR and whether UBE2C E2 is involved.

In this study, we showed that UBE2C is an oncogenic protein in the lung, supported by association studies in human lung tumor tissues, in vitro cell culture gain- or loss-of-function studies using lung cancer cell lines harboring *Kras* mutations, and an in vivo conditional KO study using a *Kras^G12D^* mouse lung tumorigenesis model. Mechanistically, we found that DEPTOR levels fluctuated with cell cycle progression and inversely correlated with UBE2C and CDH1 levels. UBE2C coupled with CDH1 to promote DEPTOR ubiquitylation and degradation. The growth suppression by *UBE2C* knockdown could be largely rescued by simultaneous *DEPTOR* knockdown in lung cancer cells. Consistently, the tumor suppression by *Ube2c* KO in the *Kras^G12D^* lung cancer model could be largely abrogated by simultaneous *Deptor* KO, indicating a causal relationship between UBE2C and DEPTOR. Taken together, our study identified an E2/E3 pair for DEPTOR ubiquitylation and degradation, and validated UBE2C as a promising target for lung cancer associated with *Kras* mutations.

## Results

### UBE2C and UBE2S are overexpressed in NSCLC, and their overexpression is associated with poor survival in LUAD patients.

To understand the role of UBE2C/UBE2S E2 conjugating enzymes in the development of lung cancer, we first searched the cBioPortal database (https://www.cbioportal.org), which is generated largely based on The Cancer Genome Atlas (TCGA), to systematically analyze the genetic changes in the *UBE2C* and *UBE2S* genes in lung cancer samples. Among the 1144 lung cancer samples analyzed, approximately 1.6% of them contained *UBE2C* changes, with a majority (1.13%) being gene amplification and approximately 2% of cases with *UBE2S* amplification (Supplemental Figure 1A; supplemental material available online with this article; https://doi.org/10.1172/JCI162434DS1). We then used a gene expression profiling dynamic analysis database (Gene Expression Profiling Interactive Analysis, GEPIA) (45) to determine the expression levels of *UBE2C* and *UBE2S* between non–small cell lung cancer (NSCLC) and normal lung tissues, and found that both genes are overexpressed in lung adenocarcinoma (LUAD) and lung squamous cell carcinoma (LUSC) tissues (Supplemental Figure 1B). We further analyzed the association of *UBE2C* and *UBE2S* levels of both mRNAs (Supplemental Figure 1C) and proteins (Supplemental Figure 1D) with survival of patients, and found a significant positive correlation between higher *UBE2C* levels and worse survival for LUAD, but not for LUSC patients (Supplemental Figure 1, C and D). On the other hand, higher *UBE2S* levels also predicted a worse outcome for LUAD, but a better survival for LUSC patients (Supplemental Figure 1, C and D). Taken together, these results imply that both E2 genes are associated with NSCLC. For the rest of study, we focused on LUAD to determine the causal roles of these 2 E2s in growth and survival of lung cancer cells in in vitro culture settings, and in in vivo lung tumorigenesis induced by mutant *Kras*.

### Knockdown of UBE2C, but not UBE2S, inhibits growth of lung cancer cells.

**Figure 1:**
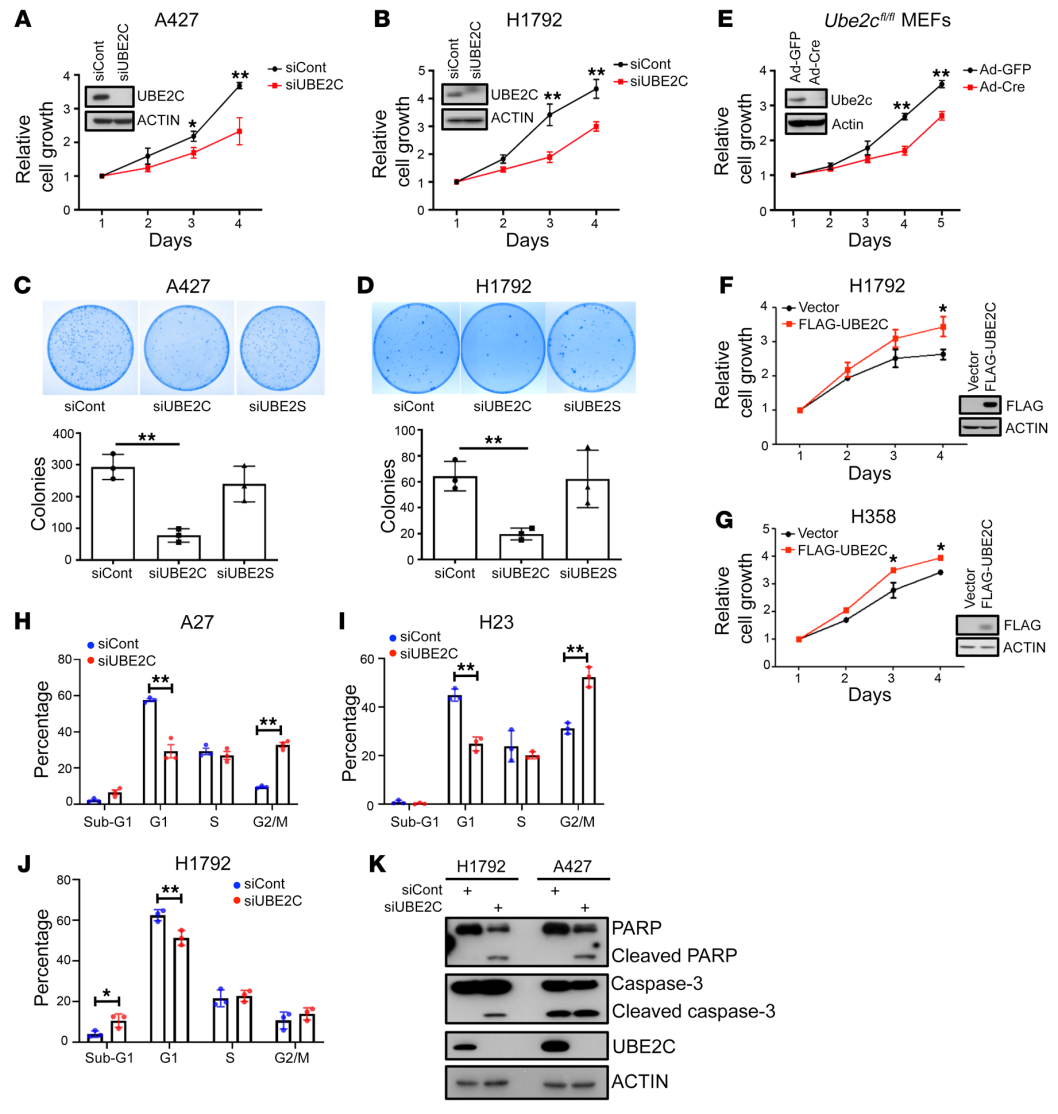
Manipulation of UBE2C, but not UBE2S, affects the proliferation and survival of lung cancer cells. (**A** and **B**) A427 (**A**) and H1792 (**B**) cells were transfected with siRNA targeting *UBE2C* or control (siCont) for 24 hours. Cells were then seeded in 96-well plates in triplicate and analyzed with a CCK-8 cell proliferation assay. (**C** and **D**) A427 (**C**) and H1792 (**D**) cells were transfected with siRNA targeting *UBE2C*, *UBE2S*, or siCont for 24 hours, followed by clonogenic survival assay. Representative pictures were taken (top) and colonies were counted and are shown as mean ± SEM (*n* = 3) (bottom). (**E**) MEFs were infected with Ad-GFP or Ad-Cre for 72 hours and analyzed by CCK-8 cell proliferation assay or IB (inset). (**F** and **G**) H1792 (**F**) and H358 (**G**) cells were transfected with plasmid expressing FLAG-tagged UBE2C or vector control for 48 hours and analyzed by CCK-8 cell proliferation assay or IB (inset). (**H**–**J**) A427 (**H**), H23 (**I**), and H1792 (**J**) cells were transfected with siRNA targeting *UBE2C* or siCont for 48 hours, followed by FACS analysis. (**K**) H1792 and A427 cells were transfected with siRNA targeting *UBE2C* or siCont for 48 hours, followed by IB with indicated antibodies. *P < 0.05; **P < 0.01 by 2-tailed Student’s *t* test (**A**, **B**, and **E**–**J**) or 1-way ANOVA test (**C** and **D**).

We next determined the causal roles of UBE2C and UBE2S in controlling the growth and survival of human LUAD cell lines harboring the *Kras^G12D^* mutation (A427) or *Kras^G12C^* mutation (H1792, H23, and H358), using siRNA-based knockdown. Indeed, *UBE2C* knockdown significantly inhibited the growth and clonal survival of both A427 and H1792 cells (Figure 1, A–D), whereas *UBE2S* knockdown had no effect on the growth of these 2 lung cancer lines (Supplemental Figure 1, E and F). Moreover, *Ube2c* deletion in mouse embryonic fibroblasts (MEFs) also suppressed cell growth (Figure 1E). Ectopic overexpression of *UBE2C* promoted the growth of lung cancer cells (Figure 1, F and G). Thus, Ube2C is essential for the growth of lung cancer cells.

To determine the nature of growth suppression, we performed fluorescence-activated cell sorting (FACS) profiling and found that *UBE2C* knockdown caused significant G_2_/M arrest in both A427 and H23 cells (Figure 1, H and I), and an increased sub-G_1_ population, indicative of apoptosis in H1792 cells (Figure 1J). Induction of apoptosis was confirmed by the cleavage of poly(ADP-ribose) polymerase (PARP) and caspase-3 (Figure 1K). Collectively, these results clearly showed that in in vitro cell culture models, *UBE2C* is essential for growth and survival of lung cancer cells harboring a mutant *Kras*.

### Inactivation of Ube2c, but not Ube2s, inhibits lung tumorigenesis induced by Kras^G12D^, and extends the lifespan of mice.

**Figure 2:**
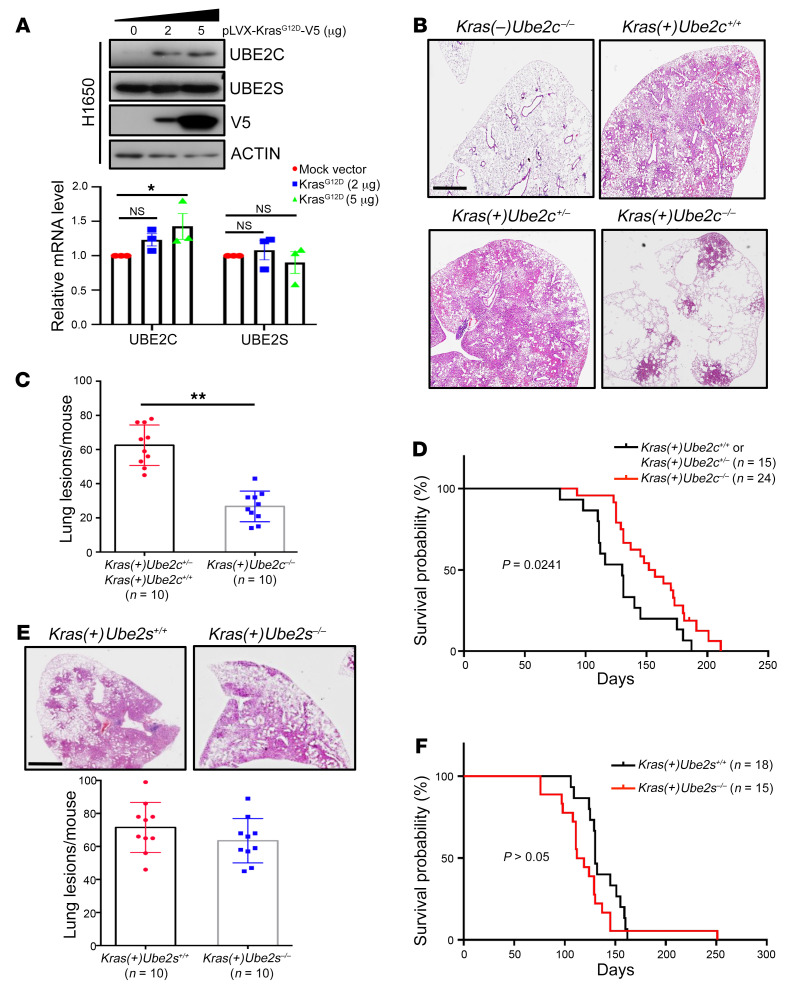
Deletion of *Ube2c*, but not *Ube2s*, inhibits lung tumorigenesis induced by *Kras^G12D^*, and extends the lifespan of mice. (**A**) H1650 cells were transfected with plasmid pLVX-Kras^G12D^-V5, followed by IB with indicated antibodies (top), or by RT-qPCR analysis (bottom). (**B**) *Ube2c* deletion remarkably reduced lung tumor burden in the *Kras^G12D^* mouse model. The lung tissues were isolated from mice with indicated genotypes, fixed, sectioned, and stained with H&E. (**C**) Quantification of mouse lung tumors in mice with indicated genotypes after Ad-Cre administration. The lesions (hyperplasia and lung tumors) in all 5 lobes of lung tissues were counted (*n* = 10 per group). (**D**) Kaplan-Meier survival curves of mice with indicated genotypes and number for up to 30 weeks after administration of Ad-Cre. (**E**) The lung tissues from mice with indicated genotypes were fixed, sectioned, and stained with H&E. (**F**) Kaplan-Meier survival curves of mice with indicated genotypes and number for up to 36 weeks after administration of Ad-Cre. *P < 0.05; **P < 0.01 by 1-way ANOVA test (**A**), 2-tailed Student’s *t* test (**C** and **E**), or the log-rank test (**D** and **F**). Scale bars: 20 μm.

These cancer-tissue association and in vitro cell-based studies suggested that *UBE2C* is a *Kras*-cooperative gene. We next examined whether UBE2C is under *Kras^G12D^* regulation, and found that indeed ectopic overexpression of *Kras^G12D^* significantly increased UBE2C at both the mRNA and protein levels without affecting UBE2S levels (Figure 2A and Supplemental Figure 2A). We then generated a mouse genetic model to further investigate the causal role of *Ube2c* in *Kras^G12D^*-driven lung cancer development. We used a well-established *LSL-Kras^G12D^* (Lox-STOP-Lox *Kras^G12D^*) lung tumorigenesis model, in which mutant *Kras^G12D^* is activated by Cre recombinase via intratracheal instillation of Cre-expressing adenovirus (Ad-Cre) to delete the STOP element, leading to sequential development of epithelial hyperplasia, adenomas, and eventually adenocarcinomas in the lung (46). To this end, we generated a *Ube2c^fl/fl^* conditional KO mouse model via Cre-driven deletion of exons 2 and 3 of the *Ube2c* allele (Supplemental Figure 2B). The *Ube2c^fl/fl^* mice were crossed with *LSL-Kras^G12D^* mice to generate compound mice with the genotypes *LSL-Kras^G12D^;Ube2^+/+^* or *LSL-Kras^G12D^;Ube2c^fl/+^* and *LSL-Kras^G12D^;Ube2c^fl/fl^*. The mice were intratracheally administered Ad-Cre to only activate *Kras^G12D^* [*Kras(+)Ube2c^+/–^* or *Kras(+)Ube2c^+/+^*, WT control group] or to simultaneously activate *Kras^G12D^* and inactivate *Ube2c* [*Kras(+)Ube2c^–/–^*, experimental group]. We also included *Kras(–)Ube2c^–/–^* mice as a negative control. Mice were euthanized 16 weeks after Cre administration. H&E staining analysis revealed that while mice with *Ube2c* deletion alone [*Kras(–)Ube2c^–/–^*] had normal lung development without any tumor formation (Figure 2B and Supplemental Figure 2C), the WT mice [*Kras(+)Ube2c^+/+^* or *Kras(+)Ube2c^+/–^*] developed, as expected, multiple lung adenomas with few adenocarcinomas (Figure 2B and Supplemental Figure 2C). Strikingly, the mice with *Ube2c* deletion [*Kras(+)Ube2c^–/–^*] had remarkably reduced lung tumor burden (Figure 2, B and C, and Supplemental Figure 2C). Thus, UBE2C is dispensable for normal lung growth, but required for lung tumorigenesis induced by *Kras^G12D^*.

We next determined the effect of UBE2C inactivation on overall survival of mice upon *Kras^G12D^* activation by comparison of the survival probability between 2 groups. Upon *Kras^G12D^* activation, WT mice had a median survival time of approximately 130 days and 100% death by 175 days, whereas the *Kras(+)Ube2c^–/–^* mice had a median survival time of approximately 150 days and 100% death by 210 days. The difference is statistically significant (*P* = 0.0241) (Figure 2D), indicating that reduced tumor burden upon *Ube2c* deletion indeed led to better animal survival.

Similarly, we also generated *Ube2s^fl/fl^* mice (Supplemental Figure 2D), and the compound *Kras(+)Ube2s^–/–^* mice also developed lung tumors but with a survival rate that was statistically indistinguishable from that of WT [*Kras(+)Ube2s^+/+^*] mice (Figure 2, E and F, and Supplemental Figure 2E). Thus, similar to the in vitro cell culture study, which showed no effect on growth of lung cancer cells upon *UBE2S* knockdown (Figure 1, C and D, and Supplemental Figure 1, E and F), this in vivo study using a lung tumor model also demonstrated that *Ube2s* is not required for lung tumorigenesis induced by *Kras^G12D^* activation.

### UBE2C differentially regulates mTORC signaling and DEPTOR levels.

**Figure 3:**
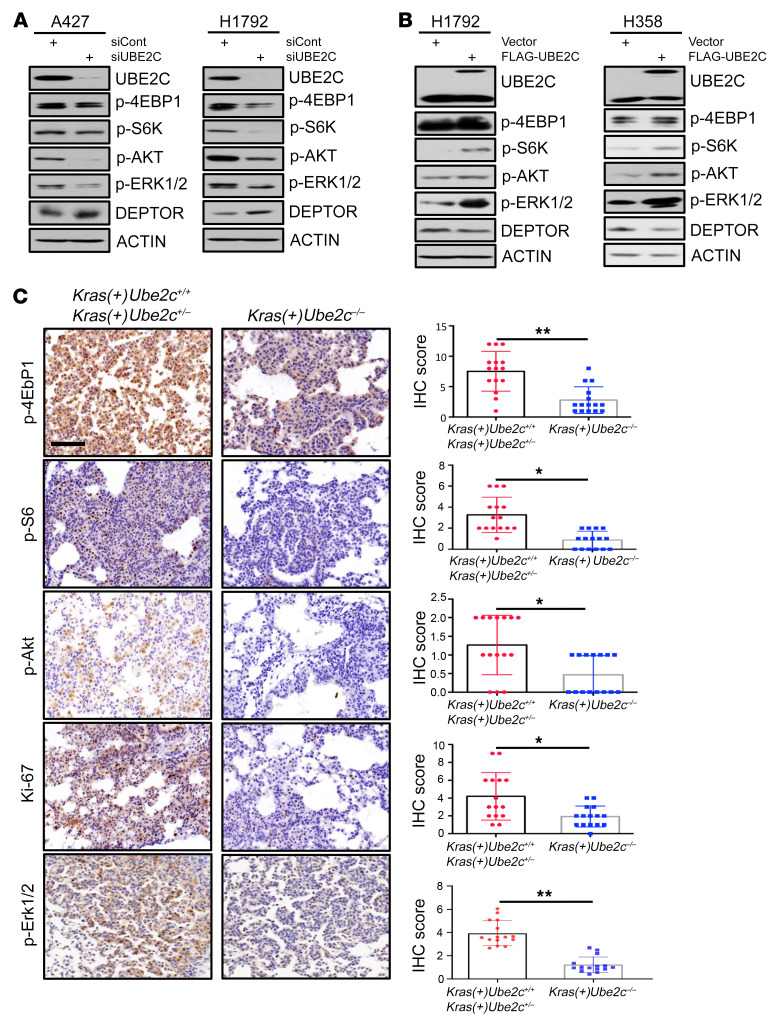
UBE2C differentially regulates mTORC signaling and DEPTOR levels. (**A**) H1792 and A427 cells were transfected with siRNA targeting *UBE2C* or siCont for 48 hours, followed by IB with indicated antibodies. (**B**) H1792 and H358 cells were transfected with plasmids of FLAG-UBE2C or vector for 48 hours, followed by IB with indicated antibodies. (**C**) *Ube2c* deletion inactivates mTORC1/2 signals. The lung tissues were isolated from mice with indicated genotypes, fixed and sectioned, and stained by IHC with the indicated antibodies. The staining quantification was analyzed by a semiquantitative immunoreactivity scoring system, as described in the Methods section. *P < 0.05, **P < 0.01 by 2-tailed Student’s *t* test (**C**). Scale bar: 50 μm.

We next investigated possible underlying mechanisms by which *UBE2C* knockdown suppressed growth and survival of lung cancer cells. It is well established that UBE2C couples with APC/C E3 ligase to promote cell cycle progression from G_2_ to M and within the M phase by targeting several regulatory protein substrates for degradation (4). Therefore, we first determined whether *UBE2C* knockdown would affect the levels of these substrates, including PLK1, cyclin B1, and securin, and found a minor, if any, effect in 4 lung cancer cell lines harboring mutant *Kras* (Supplemental Figure 3A), suggesting that these substrates are not actively involved in growth suppression induced by *UBE2C* knockdown. We then focused on the MAPK and mTORC pathways, 2 major regulatory pathways of cell growth and survival (47, 48), and found that *UBE2C* knockdown caused moderate inactivation of MAPK signaling with a moderate reduction in p-ERK1/2, but significant inactivation of mTORC1/2 signaling with a remarkable reduction in p-S6K1/p-4EBP1 (mTORC1) and p-AKT (mTORC2) in both A427 and H1792 lung cancer cells (Figure 3A). Since DEPTOR is known to inhibit both mTORC1 and mTORC2 (33), we measured DEPTOR levels and found that they were indeed increased upon *UBE2C* knockdown in both cell lines (Figure 3A), as well as in 2 additional lines of lung cancer cells (Supplemental Figure 3B) and MEFs (Supplemental Figure 3C). Consistently, ectopic expression of *UBE2C* at a level equal to or lower than endogenous levels activated both MAPK and mTORC1/2 signaling, with a moderate reduction in DEPTOR levels (Figure 3B). On the other hand, unlike *UBE2C* knockdown, *UBE2S* knockdown had no effect on MAPK and mTORC signaling in A427 cells, whereas inactivated mTORC appeared to be independent of DEPTOR in H1792 cells (Supplemental Figure 3D), largely consistent with its lack of effect on the growth of lung cancer cells upon depletion (Supplemental Figure 1, E and F).

We then extended these in vitro cell culture observations to *Kras^G12D^*-induced in vivo lung tumor tissues. Indeed, *Ube2c* deletion significantly inactivated mTORC1 and mTORC2 signaling and inhibited the proliferation rate, as reflected by remarkably reduced staining of p-4Ebp1, p-S6, p-AKT, Ki67, and p-Erk1/2 in lung tumor tissues derived from *Kras(+)Ube2c^–/–^* mice, as compared with those derived from WT mice (Figure 3C). Taken together, our results indicated that by increasing DEPTOR levels, *Ube2c* deletion inactivated mTORC signaling to inhibit *Kras^G12D^*-induced lung tumorigenesis, and suggested that UBE2C could be an effective therapeutic target for management of lung cancer associated with *Kras* mutation.

### DEPTOR is a cell cycle regulatory protein, controlled by the UBE2C/CDH1 axis.

**Figure 4:**
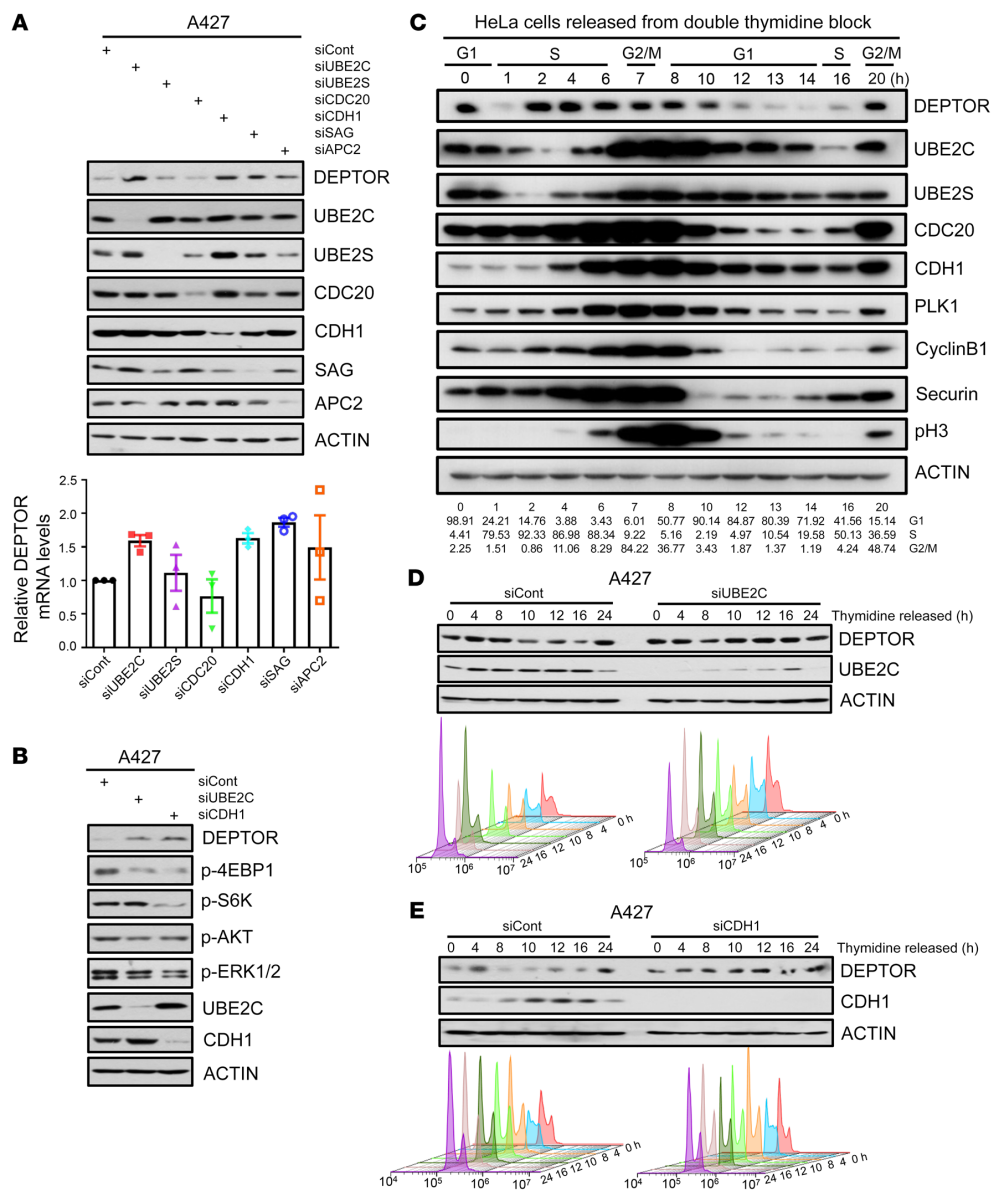
DEPTOR is a cell cycle regulatory protein, controlled by the UBE2C/CDH1 axis. (**A**) Knockdown of *UBE2C*, *CDH1*, *SAG*, and *APC2* caused DEPTOR accumulation at the protein level, but not at the mRNA level. A427 cells were transfected with indicated siRNAs followed by IB with the indicated Abs (top), or by RT-qPCR analysis (bottom). (**B**) Knockdown of *CDH1* and *UBE2C* caused the accumulation of DEPTOR and inactivation of mTORC1/2. A427 cells were transfected with indicated siRNAs followed by IB with the indicated Abs. (**C**) DEPTOR levels fluctuated during the cell cycle. HeLa cells were arrested at the G_1_/S phase by double thymidine block (treatment with 2 mM thymidine for 14 hours and release for 9 hours, and then treatment with 2 mM thymidine for another 14 hours), and then released into the normal cell cycle. Cells were harvested at indicated times and analyzed by IB. (**D** and **E**) Knockdown of *UBE2C* and *CDH1* abrogated the cell cycle fluctuation of DEPTOR. A427 cells were transfected with siRNA targeting *UBE2C* (**D**), *CDH1* (**E**), or siCont for 24 hours, and then cells were synchronized at the G_1_/S phase by thymidine block and released for the indicated time points. Cells were harvested for IB analysis with indicated Abs (top) or FACS analysis (bottom).

UBE2C and UBE2S are not only canonical E2s coupling with APC/C E3 ligase, but also act as E2s to work with SAG-E3 ligase for substrate ubiquitylation, as we recently reported (7, 8). We next determined the potential effect of manipulating APC/C components and SAG on DEPTOR levels. Knockdown of *CDH1*, *APC2* and *SAG*, but not of *UBE2S* and *CDC20*, increased DEPTOR protein levels with little or no effect on *DEPTOR* mRNA levels in both lung cancer cell lines (Figure 4A and Supplemental Figure 4A). Since we have previously shown that *SAG* knockdown causes DEPTOR accumulation to block mTORC signaling in prostate cancer cells (49), we focused our study on CDH1. Like *UBE2C* knockdown, *CDH1* knockdown also inactivated mTORC1/2 signaling in all 3 cell lines tested without affecting the MAPK signaling in 2 cell lines (Figure 4B and Supplemental Figure 4B), suggesting that APC/C^CDH1^ E3 couples with UBE2C E2 to control the abundance of DEPTOR, thus regulating mTORC1/2 signaling.

Given that the levels of most substrates of APC/C fluctuated during cell cycle progression, we wondered whether that is the case for DEPTOR. To this end, we arrested cells at the G_1_/S boundary by double thymidine blockage and then collected cells at various time points after releasing, followed by immunoblotting (IB) for the levels of DEPTOR, along with UBE2C/2S, CDH1, CDC20, and a few known substrates of APC/C E3, including PLK1, cyclin B1, and securin. Interestingly, the levels of DEPTOR, like all other proteins tested and known to fluctuate with the cell cycle, indeed fluctuated during cell cycle progression. DEPTOR levels were rather high at the S and G_2_/M phases, but lowest at the G_1_ phase in a roughly reverse correlation with the levels of UBE2C and CDH1 (Figure 4C). We further confirmed that cell cycle–dependent fluctuation of DEPTOR levels was dependent on UBE2C or CDH1, since it was completely abrogated by the knockdown of either of them (Figure 4, D and E, and Supplemental Figure 4, C and D). However, it was independent of β-TrCP, another SCF type of E3 known to ubiquitylate and degrade DEPTOR in response to serum stimulation (41–43), since the DEPTOR levels in cells with *β-TrCP* knockdown also fluctuated during cell cycle progression (Supplemental Figure 4E). Collectively, these results clearly demonstrated that DEPTOR is regulation by, and could be a new substrate of, the UBE2C-APC/C^CDH1^ E2/E3 pair.

### DEPTOR is a substrate of the UBE2C-APC/C^CDH1^ E2-E3 complex.

**Figure 5:**
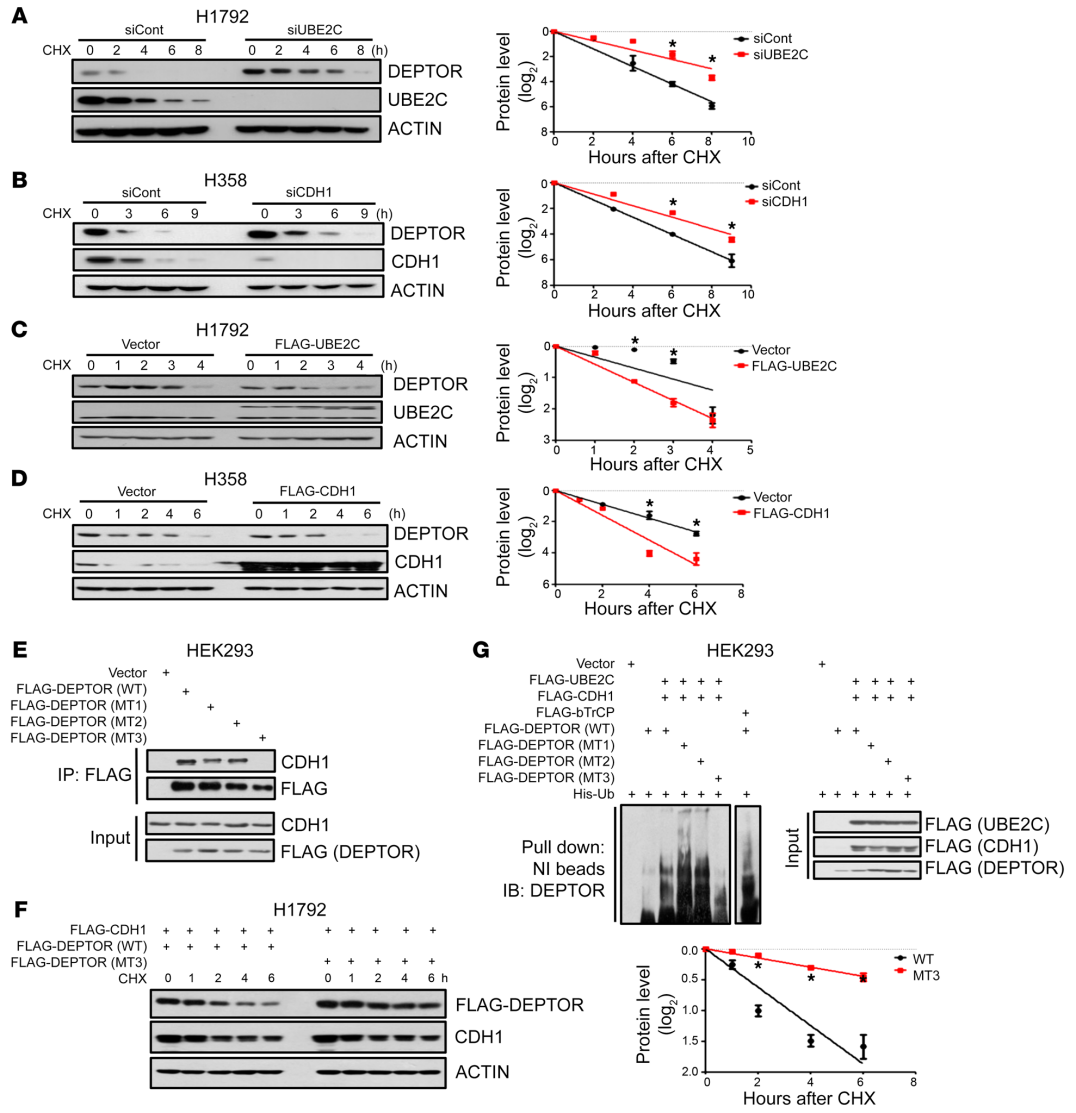
DEPTOR is a substrate of the UBE2C-APC/C^CDH1^ E2-E3 complex. (**A** and **B**) H1792 (**A**) and H358 (**B**) cells were transfected with indicated siRNAs for 48 hours, and cells were then treated with cycloheximide (CHX) for the indicated time periods and harvested for IB (left). The band density was quantified using ImageJ software (NIH), and the decay curves are shown (right). (**C** and **D**) H1792 (**C**) and H358 (**D**) cells were transfected with the indicated plasmids for 48 hours, and then treated with CHX for the indicated time periods and harvested for IB (left). The decay curves are shown (right). (**E**) HEK293 cells, transfected with indicated plasmids, were pulled down with anti-FLAG beads followed by IB with the indicated Abs. (**F**) H1792 cells were transfected with the indicated plasmids for 48 hours and then treated with CHX for the indicated time periods and harvested for IB (left). The decay curves are shown (right). (**G**) HEK293 cells were transfected with the indicated combination of plasmids for 48 hours, cell lysates were harvested after 8-hour treatment with 10 mM MG132, pull downed with Ni-NTA beads in 8 M urea, resolved by PAGE, and blotted with anti-DEPTOR Ab. *P < 0.05 by 1-way ANOVA test (**A**–**D** and **F**).

We next characterized UBE2C-APC/C^CDH1^ as a bona fide E2-E3 for DEPTOR. We first confirmed a direct binding between CDH1 and DEPTOR by reciprocal immunoprecipitation (IP) assays (Supplemental Figure 5A). In multiple lung cancer cell lines, knockdown of either *UBE2C* or *CDH1* significantly extended the protein half-life of DEPTOR (Figure 5, A and B, and Supplemental Figure 5, B and C), while ectopic overexpression of *UBE2C* or *CDH1* shortened the protein half-life of DEPTOR (Figure 5, C and D, and Supplemental Figure 5, D and E).

The D-box (RXXLXXXXD/E/D) is known as the substrate recognition motif of CDH1 (50). We searched the DEPTOR protein sequence and identified 3 evolutionarily conserved D-box motifs (designated as D1, D2, and D3) (Supplemental Figure 5F). We then generated AXXA double D-box mutants to replace key residues of Arg (R) and Leu (L) with Ala (A) residues, designated as MT1, MT2, and MT3 (Supplemental Figure 5F). We found that like WT DEPTOR, MT1 and MT2 mutants retained CDH1 binding, whereas MT3 completely lost CDH1 binding (Figure 5E), indicating that D3 is the primary degron motif required for CDH1 binding. Consistently, ectopic overexpression of *CDH1* shortened the protein half-life of WT as well as the MT1 and MT2 DEPTOR mutants, but had no effect on the MT3 mutant (Figure 5F and Supplemental Figure 5G). Finally, we conducted in vivo ubiquitylation assays, using β-TrCP as a positive control, and found that ectopically expressed *CDH1* and *UBE2C* significantly promoted the polyubiquitylation of WT and the MT1 and MT2 DEPTOR mutants, but not the MT3 mutant (Figure 5G). Taken together, these results clearly indicate that DEPTOR is indeed a substrate of APC/C^CDH1^, subjected to its ubiquitylation in a manner dependent on the D3-box motif.

### DEPTOR knockdown rescues the phenotypes induced by UBE2C knockdown in in vitro cell culture models.

As a natural mTORC1/2 inhibitor, DEPTOR is generally considered a tumor suppressor in most cases (24–32). We analyzed the prognostic value of DEPTOR in lung cancer by using the Kaplan-Meier Plotter database (https://kmplot.com) and the Human Protein Altas (https://www.proteinatlas.org), and found that higher levels of DEPTOR mRNA and protein were associated with better prognosis for LUAD, but not for LUSC (Supplemental Figure 6, A and B), indicating an opposite association between DEPTOR and UBE2C with the survival of LUAD patients.

**Figure 6:**
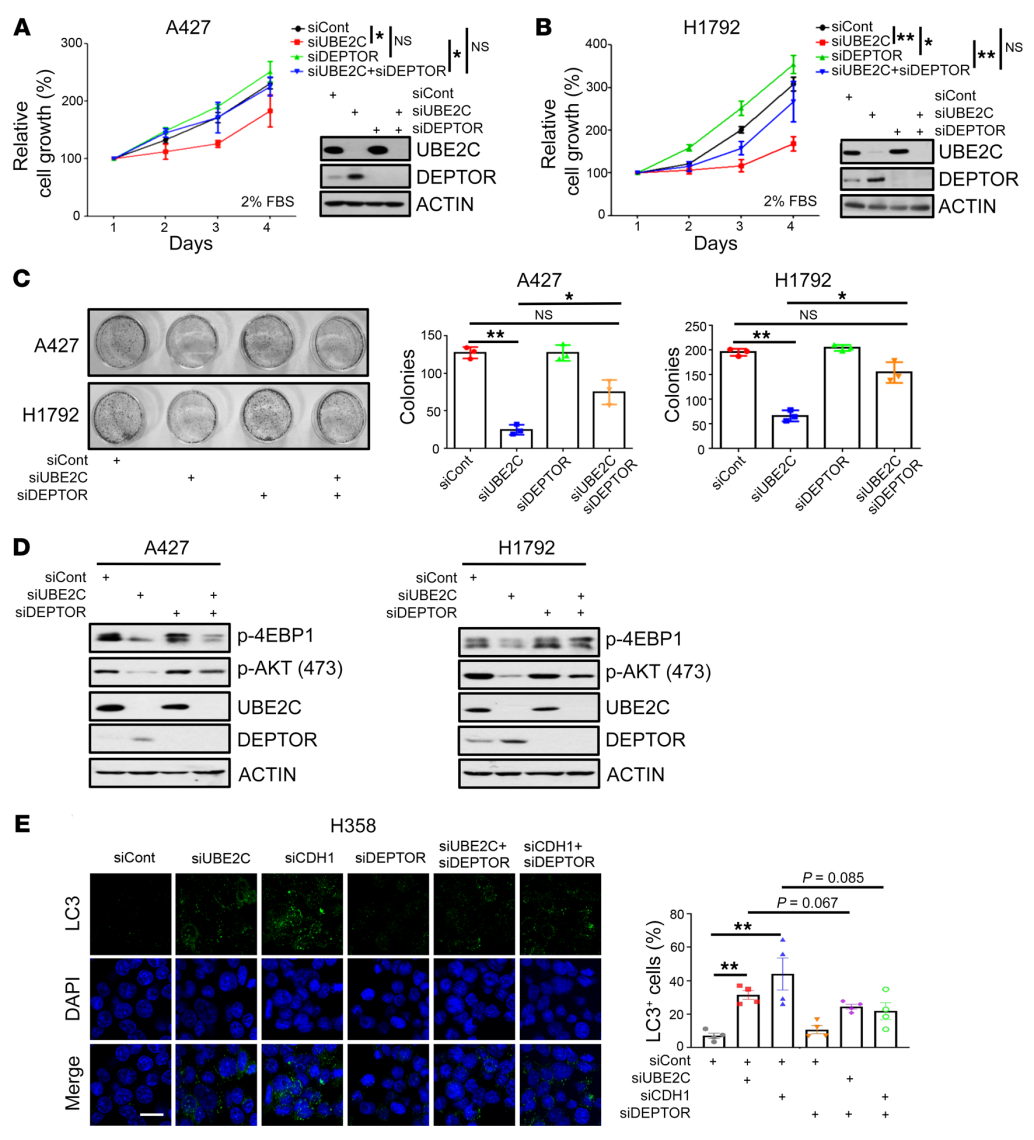
*DEPTOR* knockdown rescues the phenotypes induced by *UBE2C* knockdown in lung cancer cells. (**A** and **B**) A427 (**A**) and H1792 (**B**) cells were transfected with indicated siRNAs for 24 hours; one portion of cells was analyzed by IB (inset), and the other portion was seeded in 96-well plates with medium containing 2% FBS in triplicate and analyzed by CCK-8 cell proliferation assay on the indicated days. (**C**) A427 and H1792 cells were transfected with the indicated siRNAs for 24 hours, followed by clonogenic survival assay. A representative dish from each indicated group was photographed (left) and colonies were counted and are shown as mean ± SEM (*n* = 3) (right). (**D**) H1792 and A427 cells were transfected with indicated siRNAs for 48 hours, followed by IB with indicated antibodies. (**E**) Knockdown of *UBE2C* or *CDH1* induced autophagy, which was rescued by *DEPTOR* knockdown. H358 cell were transfected with indicated siRNAs for 48 hours and stained with the indicated Abs, followed by photography under a fluorescence microscope (left). Data are the mean ± SEM of 4 independent experiments (right). *P < 0.05; **P < 0.01 by 1-way ANOVA test (**A**–**C** and **E**). Scale bar: 20 μm.

To investigate whether DEPTOR accumulation played a causal role in growth suppression induced by *UBE2C* knockdown, we simultaneously knocked down *DEPTOR* and *UBE2C*, and observed that under nutrient-enriched conditions (10% fetal bovine serum, FBS), *DEPTOR* knockdown itself had no effect on cell growth, nor rescued growth suppression induced by *UBE2C* knockdown (Supplemental Figure 6, C and D). However, under nutrient-deprived culture conditions (2% FBS), *DEPTOR* knockdown alone promoted cell growth and partially rescued the growth-suppressing effect of *UBE2C* knockdown (Figure 6, A and B). A clonogenic survival assay also showed a partial rescue effect by *DEPTOR* knockdown (Figure 6C). *DEPTOR* knockdown also rescued inactivation of mTORC1/2 by *UBE2C* knockdown (Figure 6D). Since mTOR signal is a well-known negative regulator of autophagy (51), we then determined the effect of the UBE2C/CDH1/DEPTOR axis on autophagy and found that knockdown of either *UBE2C* or *CDH1* induced autophagy, as demonstrated by enhanced LC3 immunofluorescent staining, LC3-I to LC3-II conversion, and p62 degradation in both H358 and H1975 lung cancer cells, which were partially rescued by simultaneous knockdown of *DEPTOR* (Figure 6E and Supplemental Figure 6, E–G). These findings suggested that DEPTOR accumulation, followed by mTORC1/2 inactivation, was causally related at least in part to the inhibition of growth and survival and induction of autophagy triggered by *UBE2C* knockdown in lung cancer cells.

### Deptor KO rescues the phenotypes induced by Ube2c KO in the Kras^G12D^ lung cancer model.

Given that *DEPTOR* knockdown rescued the phenotypes induced by *UBE2C* knockdown in in vitro cell culture models, we then determined in vivo whether *Deptor* KO could rescue the suppression of lung tumorigenesis by *Ube2c* KO in the *Kras^G12D^* mouse lung cancer model. To this end, we introduced a *Deptor*-KO mouse model by Cre-driven deletion of exons 6 and 7 of the *Deptor* allele, as previously described (24). By proper mating with *LSL*-*Kras^G12D^;Ube2c^fl/fl^* mice, we ultimately generated 3 types of compound mice with the following genotypes: (a) *Kras(+)Ube2c^+/–^;Deptor^+/–^* (WT); (b) *Kras(+)Ube2c^–/–^;Deptor^+/–^* (*Ube2c*-null); and (c) *Kras(+)Ube2c^–/–^;Deptor^–/–^* (*Ube2c* and *Deptor* double null). Note that mice with heterozygous deletion of *Ube2c* (*Ube2c^+/–^*) or *Deptor* (*Deptor^+/–^*) have no phenotypes, and *Deptor* deletion was previously shown to accelerate the formation of lung tumors in a *Kras^G12D^*/*p53*-null mouse model (26).

**Figure 7:**
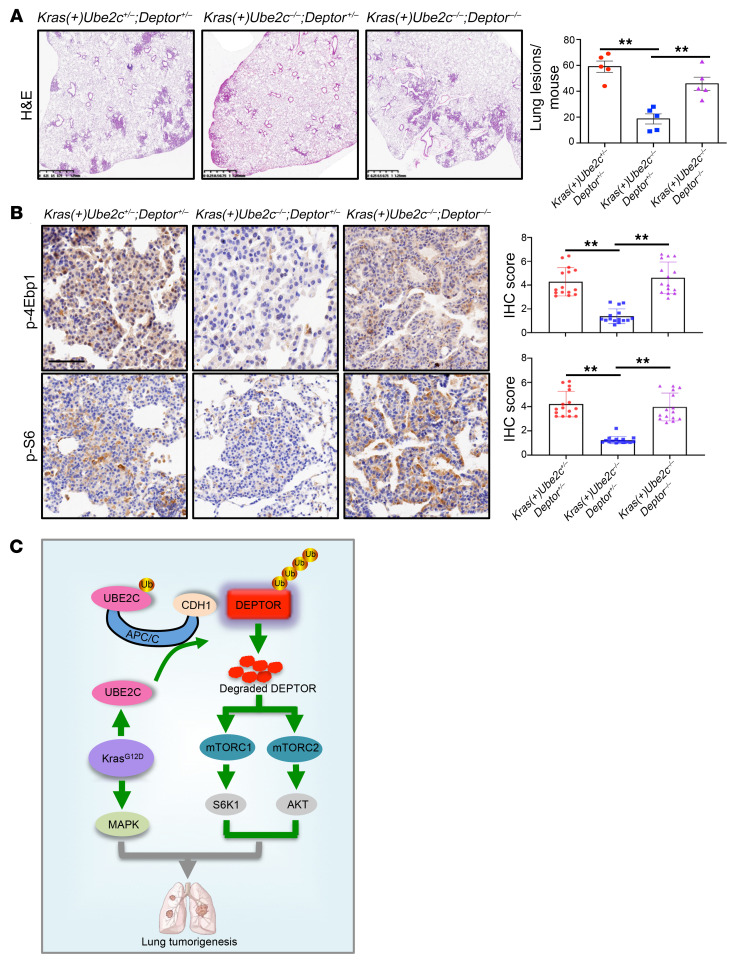
*Deptor* KO rescues the *Ube2c*-KO phenotypes in the *Kras^G12D^* tumor model. (**A**) Lung tissues were isolated from mice with 3 indicated genotypes, fixed, sectioned, and stained with H&E (left). Quantification of mouse lung tumors developed in mice with indicated genotypes after Ad-Cre administration (right). The lesions (hyperplasia and lung tumors) in all 5 lobes of lung tissues were counted (*n* = 5 per group). (**B**) Lung tumor sections were stained by IHC with the indicated antibodies. The staining quantification was analyzed by a semiquantitative immunoreactivity scoring system, as described in the Methods section. **P < 0.01 by 1-way ANOVA test (**A** and **B**). Scale bars: 1.25 mm (**A**) and 50 μm (**B**). (**C**) Working model: During *Kras^G12D^*-driven lung tumorigenesis, *Kras^G12D^* induces UBE2C expression. Aberrantly activated UBE2C then promotes the ubiquitylation and degradation of tumor suppressor DEPTOR by coupling with APC/C^CDH1^ E3 ligase, leading to activation of mTORC1 (p-S6K1) and mTORC2 (p-AKT), which cooperate with Kras^G12D^ to promote lung tumorigenesis.

After Ad-Cre administration to activate *Kras^G12D^* and delete *Ube2c* alone or in combination with *Deptor*, we performed IB analysis of mouse lung tissues and confirmed DEPTOR accumulation in *Kras(+)Ube2c^–/–^;Deptor^+/–^*, and DEPTOR depletion in *Kras(+)Ube2c^–/–^;Deptor^–/–^* mice (Supplemental Figure 7A). H&E staining showed clearly that tumor suppression by *Ube2c* deletion was completely rescued by simultaneous *Deptor* deletion in double-null mice with tumor burdens, similar to *Kras^G12D^* WT mice (Figure 7A and Supplemental Figure 7B). Immunohistochemistry (IHC) showed that mTORC1/2 inactivation by *Ube2c* deletion was also rescued by simultaneous deletion of *Deptor*, as reflected by increased staining of p-4Ebp1, p-S6, p-AKT, and Ki67 in the lung tumor tissues of double-null mice (Figure 7B and Supplemental Figure 7C). Collectively, these results demonstrated that the tumor-suppression phenotype resulting from *Ube2c* deletion was mainly mediated via DEPTOR accumulation and subsequent inactivation of mTORC1/2 signaling in the *Kras^G12D^* lung tumorigenesis model.

## Discussion

Although previous studies, using in vitro cell culture settings, have shown the growth-promoting and autophagy-suppressing effects of UBE2C in lung cancer cells (19, 52), to the best of our knowledge no in vivo study using a *Ube2c* conditional KO mouse model has been conducted to understand the role of UBE2C during lung tumorigenesis. In this study, using both in vitro cell culture and in vivo mouse models, we showed that *Ube2c* is a *Kras*-cooperative gene during lung tumorigenesis, and elucidated its mechanism of action by targeting DEPTOR for degradation, thus activating mTORC signaling to inhibit autophagy. Our study is consistent with a previous cell culture–based study, reporting that UBE2C suppressed autophagy in lung cancer cells via an unknown mechanism (19).

We first performed an association study using TCGA database containing more than 400 lung cancer cases, and found that overexpression of either UBE2C or UBE2S is correlated with the survival of LUAD patients. However, the association study did not reveal the cause or consequence of UBE2C/UBE2S overexpression in lung cancer development. To address this, we first used an in vitro cell culture system and confirmed that UBE2C, but not UBE2S, is essential for the growth and survival of lung cancer cells harboring a mutant *Kras*. We then used in vivo conditional KO mouse models and showed that deletion of *Ube2c*, but not *Ube2s*, significantly inhibited lung tumorigenesis induced by *Kras^G12D^*.

What is the molecular mechanism by which UBE2C acts as a growth-essential and *Kras*-cooperative gene in lung cancer? Previous studies have shown that aberrant activation of UBE2C rewires multiple downstream molecular cascades during tumorigenesis (12, 53). Given that UBE2C is known to couple with APC/C E3 to promote ubiquitylation and degradation of several cell cycle regulators that govern the progression from G_2_ to M and within the M phase, including PLK1, cyclin B1, and securin (22, 54, 55), we first investigated the involvement of these substrates, but found no significant accumulation upon *UBE2C* knockdown in multiple lung cancer cell lines harboring a mutant *Kras*. However, we found a significant accumulation of DEPTOR, a tumor suppressor and a naturally occurring inhibitor of mTORC1/2, implying that the DEPTOR/mTORC pathway could be involved in the growth-suppressing effect of *UBE2C* knockdown. Subsequent studies revealed that UBE2C likely regulates DEPTOR/mTORC signaling, since increased levels of DEPTOR and decreased levels of p-S6K1/p-4EBP1 (mTORC1) and p-AKT (mTORC2) were evident in *UBE2C*-knockdown cells. mTORC signals are negative regulators of autophagy (51), and UBE2C-mediated autophagy repression contributes to malignant phenotypes of lung cancer cells (19), suggesting that autophagy modulation via the DEPTOR/mTORC axis could be actively involved in the growth-suppressing effect of *UBE2C* knockdown as well. More importantly, simultaneous knockdown of *DEPTOR* notably rescued the induction of autophagy and inhibition of growth and survival by *UBE2C* knockdown, demonstrating that DEPTOR accumulation is causally related at least in part to the effect of *UBE2C* knockdown. However, it should be noted that other pathways are likely involved, given the observation of partial rescue by *DEPTOR* knockdown in lung cancer cells.

Another interesting finding we made in this study is that the DEPTOR levels fluctuated during cell cycle progression, with the lowest level at the G_1_ phase when APC/C^CDH1^ is in a highly active state (4). Moreover, this fluctuation is dependent on UBE2C and CDH1, but not β-TrCP, a known E3 for DEPTOR (41–43), suggesting that DEPTOR’s turnover is likely controlled by UBE2C-APC/C^CDH1^. This notion is further supported by the observation that DEPTOR levels were increased in lung cancer cell lines upon *UBE2C* knockdown, and in lung tumor tissues derived from *Ube2c*-deleted mice.

We then characterized DEPTOR as a bona fide substrate of UBE2C-APC/C^CDH1^ with the following lines of supporting evidence: (a) DEPTOR levels are negatively regulated by UBE2C and CDH1, (b) DEPTOR protein half-life is negatively regulated by UBE2C and CDH1, and (c) DEPTOR binds to CDH1 and is subject to CDH1-mediated polyubiquitylation and half-life shortening in a manner dependent on a D-box motif in DEPTOR. Thus, we believe that APC/C^CDH1^ is a newly identified E3 ligase, in addition to β-TrCP (41–43) and SAG (49), that promotes ubiquitylation and degradation of DEPTOR in a cell cycle–dependent manner.

*Kras* mutations are found in 20% to 25% of LUAD in Western countries and in 10%–15% of cases in Asia (56). The *Kras^G12D^*-driven mouse lung tumorigenesis model recapitulates nicely the entire process of human lung tumorigenesis with sequential formation of lesions such as hyperplasia, adenoma, and eventually adenocarcinoma (46, 57). In this study, we generated *Ube2c* and *Ube2s* conditional KO mouse models (*Ube2c^fl/fl^* and *Ube2s^fl/fl^*) and combined them individually with the *LSL-Kras^G12D^* model to study the role of UBE2C/UBE2S in lung tumorigenesis. We found that KO in the lung of *Ube2c*, but not *Ube2s*, substantially reduced the tumor burden and prolonged mouse lifespan, indicating that *Ube2c* is a *Kras*-cooperative gene, largely required for *Kras*-induced lung tumorigenesis. Interestingly, we found that *Kras^G12D^* positively regulates UBE2C expression, since ectopic overexpression of *Kras^G12D^* significantly increased UBE2C at both mRNA and protein levels (Figure 2A and Supplemental Figure 2A). Furthermore, the tumor-suppression effect of *Ube2c* KO is achieved largely by accumulated DEPTOR as a substrate of UBE2C. This causal role of DEPTOR is fully demonstrated by an in vivo rescue experiment, in which suppression of tumor growth by *Ube2c* KO is fully abrogated by simultaneous *Deptor* KO. Thus, our study clearly demonstrated that the UBE2C/DEPTOR axis is an oncogene-tumor suppressor cascade that regulates lung tumorigenesis triggered by *Kras^G12D^*.

In summary, our study showed that *UBE2C* is a growth-essential gene in lung cancer cells harboring a *Kras* mutation, and *Ube2c* is required for lung tumorigenesis induced by *Kras^G12D^*. Our study fits the following working model: During *Kras^G12D^*-induced lung tumorigenesis, UBE2C is upregulated to promote ubiquitylation and degradation of the tumor suppressor DEPTOR by coupling with APC/C^Cdh1^ E3 ligase. As such, the mTORC signals (S6K1 and AKT) are activated to cooperate with *Kras^G12D^* (MAPK) for lung tumorigenesis (Figure 7C). Targeting UBE2C, therefore, could be a promising strategy for the treatment of lung cancer associated with *Kras* mutations.

## Methods

### TCGA data analysis.

The cBioPortal database (https://www.cbioportal.org/) was used to analyze the gene changes in lung cancer samples. The Gene Expression Profiling Interactive Analysis (GEPIA, http://gepia.cancer-pku.cn/) platform was used to determinate the gene expression differences between lung cancer and normal lung tissues. The Kaplan-Meier Plotter database (https://kmplot.com) (58) and the Human Protein Altas (https://www.proteinatlas.org) were used to analyze the correlation between the mRNA/protein expression levels and the prognosis of lung cancer patients.

### Cell culture.

Human embryonic kidney 293 (HEK293) cells, lung cancer A427 cells, and cervical carcinoma HeLa cells were purchased from American Type Culture Collection (ATCC) and maintained in Dulbecco’s modified Eagle’s medium (DMEM) containing 10% (v/v) FBS and 1% penicillin-streptomycin. Lung cancer H358, H23, H1792, H1975, and H1650 cells were purchased from ATCC and maintained in RPMI 1640 medium with 10% FBS and 1% penicillin-streptomycin. Human bronchial epithelial Beas-2B cells were sourced from ATCC and were grown in BEGM Bronchial Epithelial Cell Growth Medium (Lonza Group Ltd.). All cells were cultured in a 37°C humidified incubator with 5% CO_2_.

### Plasmids and siRNAs.

The plasmid constructs expressing FLAG-UBE2C, FLAG-CDH1, and FLAG-DEPTOR were maintained in the Sun lab. The D-box–mutant DEPTOR plasmid was constructed by Hangzhou Regene Biological Technology Co., Ltd. The plasmid construct pLVX-Kras^G12D^-V5 was obtained from Zhimin Lu’s Lab in the Institute of Translational Medicine, Zhejiang University School of Medicine (Hangzhou, China), and the viral construct sh-Kras^G12D^-V5 was constructed by OBiO Technology Corp., Ltd. The siRNA oligonucleotides were obtained from Genepharma, and their sequences are as follows: UBE2C siRNA, 5′-CCUGCAAGAAACCUACUCA-3′; UBE2S siRNA, 5′-CCTCCAACTCTGTCTCTAA-3′; CDH1 siRNA, 5′-UGAGAAGUCUCCCAGUCAG-3′; CDC20 siRNA, 5′-CGGCAGGACUCCGGGCCGA-3′; DEPTOR siRNA, 5′-GCCATGACAATCGGAAATCTA-3′; SAG siRNA, 5′-CCTGTGGGTGAAACAGAACAA-3′; APC2 siRNA, 5′-TGCGCGGAGTCTTGTTCTTTA-3′.

### Ectopic expression and siRNA knockdown.

Various viral constructs, plasmid constructs, and siRNA oligonucleotides were transfected using PolyJet In Vitro DNA Transfection Reagent (SignaGen Laboratories) or Genemute siRNA Transfection Reagent (Invitrogen), according to the manufacturers’ instructions.

### FACS analysis.

After siRNA transfection for 48 hours, cells were collected by trypsinization and fixed in ice-cold 75% ethanol for at least 12 hours. Cells were then stained with propidium iodide and analyzed by Cytoflex flow cytometer (Beckman).

### Cell proliferation and clonogenic survival assay.

Cell proliferation was measured using Cell Counting Kit-8 (CCK-8, MedChem Express) according to the manufacturer’s instructions. For clonogenic survival assays, 500–1000 cells were seeded into 35-mm dishes in triplicate and grown for 14 days. The colonies were fixed, stained, and counted for analysis.

### IB and co-IP.

IB and co-IP assays were performed as described previously (8). Briefly, cells were lysed in an IP lysis buffer containing 50 mM Tris-HCl (pH 8.0), 120 mM NaCl, 1% Triton X-100, 1 mM EDTA, and complete protease inhibitor cocktail (Complete Mini, Roche). Supernatants were saved for direct IB after centrifugation. For co-IP, 1–2 mg protein lysate was incubated with bead-conjugated anti-FLAG or the appropriate antibody (Ab) in a rotating incubator overnight at 4°C, followed by a 2-hour incubation with Protein A–Sepharose beads (GE Healthcare). Then, the immunocomplexes were washed 5 times with IP lysis buffer before being resolved by SDS-PAGE and analyzed by IB. Abs against the following proteins were used: DEPTOR (11816, Cell Signaling Technology), p-AKT S473 (4060, Cell Signaling Technology), p-S6K1 T389 (9234, Cell Signaling Technology), p-ERK1/2 (4376, Cell Signaling Technology), PARP (9532, Cell Signaling Technology), caspase 3 (9662, Cell Signaling Technology), p-4EBP1 (2885, Cell Signaling Technology), β-catenin (8480, Cell Signaling Technology), CDH1 (C7588, Sigma-Aldrich), PLK1 (sc-17783, Santa Cruz Biotechnology), cyclin B1 (12231P, Cell Signaling Technology), securin (ab3305, Abcam), UBE2C (271050, Santa Cruz Biotechnology), UBE2S (11878, Cell Signaling Technology), V5-Tag (13202, Cell Signaling Technology), and β-actin (A5441, Sigma-Aldrich). See complete unedited blots in the supplemental material.

### Quantitative real-time reverse transcription PCR.

RNA was isolated using TRIzol reagent (Invitrogen) and then transcribed into complementary DNA using SuperScript III reagent (Invitrogen). Quantitative real-time reverse transcription PCR (RT-qPCR) was performed using SYBR Green (RR420B, Takara Bio) on a 7900HT Real-Time PCR System (Applied Biosystems) using the following primers: DEPTOR forward, 5′-CCTACCCAAACTGTTTTGTCGC-3′; and DEPTOR reverse, 5′-CGGTCTGCTAATTTCTGCATGAG-3′. The housekeeping gene *GAPDH* was used as an internal control.

### In vivo ubiquitylation assay.

HEK293 cells were cotransfected with indicated plasmids for 48 hours and then treated with 20 μM MG132 (HY-13259, MedChem Express) for 5 hours before harvesting. Cells were lysed in 6 M guanidinium denaturing solution and sonicated. The lysates were incubated with nickel–nitrilotriacetic acid (Ni-NTA) beads (QIAGEN) for pulling down, followed by IB using indicated antibodies.

### Immunofluorescent staining.

Cells were fixed in methanol for 7 minutes at –20°C, and then blocked with PBS buffer containing 2.5% BSA and 0.1% Triton X-100 for 1 hour at room temperature. Cells were incubated with primary Ab at 4°C overnight, and then incubated with appropriate secondary Ab conjugated to Alexa Fluor 488 (Molecular Probes) for 1 hour at room temperature. Cellular nuclei were stained with DAPI. Slides were examined under a Nikon A1-Ti microscope (objective magnification, ×60) for punctate vesicle structures of LC3 and images were processed with NIS-Elements software (Nikon). The percentages of cells undergoing autophagy were plotted in a bar graph with the mean ± SEM from 3 independent experiments.

### Generation of conditional KO mice and PCR-based genotyping.

Both *Ube2c* and *Ube2s* conditional KO mice were generated using the Cas9/RNA system gene-targeting technology from GemPharmatech. For genotyping, genomic DNA was isolated from the tips of mouse tails and genotyped using the primer set Ube2c-F (5′-CTGTGGGCAAGCGGTGAGTG-3′) and Ube2c-R (5′-GGTTCAGCTCTGGCACTCAA-3′) to detect floxed (280 bp) and WT (191 bp) alleles. The primer set Ube2s-F (5′-GTATGCCAGGGGATCTGAAACAC-3′) and Ube2s-R (5′-CTCAGCATTATAGGCCAGTCACCTG-3′) was used to detect floxed (297 bp) and WT (204 bp) alleles. The *Deptor*-KO mouse model by Cre-driven deletion of exons 6 and 7 of the *Deptor* allele (24) was provided by Yongchao Zhao from the Institute of Translational Medicine, Zhejiang University School of Medicine (Hangzhou, China).

### Generation of MEFs.

MEFs were generated from E13.5 embryos with indicated genotypes as described in our previous studies (59, 60), and maintained in DMEM with 15% FBS, 2 mM L-glutamine, and 0.1 mM MEM nonessential amino acids at 37°C in a 5% CO_2_ humidified chamber.

### Ad-Cre infection of mouse lung.

To activate *Kras^G12D^* and delete *Ube2c/Ube2s* in mouse lung, transtracheal administration of Ad-Cre was performed as previously described (46). Briefly, 6- to 8-week-old mice were anesthetized with isoflurane via a gas chamber. Ad-Cre at a dose of 3 × 10^7^ PFU in a total volume of 100 μL as CaP_i_ coprecipitates was loaded in a gel-loading tip and administered transtracheally.

### IHC.

Mouse lung tissues were harvested after 10% formalin perfusion, and then fixed in 10% formalin and embedded in paraffin. Sections (5 μm thick) were cut for H&E staining and IHC. The ABC Vectastain Kit (Vector Laboratories) with Abs against p-AKT (4060, Cell Signaling Technology), p-4EBP1 (2855, Cell Signaling Technology), p-S6 (S235/236) (4858, Cell Signaling Technology), and Ki67 (ab16667, Abcam) were used for staining, and then scanned by an Aperio Whole Slide Scanner.

For quantitative evaluation, at least 5 random fields of each lobe of lung tissues were photographed at ×20 magnification and were then analyzed and calculated using a semiquantitative immunoreactivity scoring system. Specifically, stained tissues were classified into 4 groups according to the staining intensity as follows: 0, negative; 1, weak; 2, moderate; and 3, strong. The proportion scores of indicated protein expression depending on the percentage of positive cells were classified as follows: 0, 0%; 1, ≤10%; 2, 11%–50%; 3, 51%–80%; and 4, ≥81%. The total scores were calculated by multiplying the intensity score by the proportion score (61).

### Statistics.

The significance of the difference between 2 experimental groups was determined by 2-tailed Student’s *t* test, and multiple group comparisons were analyzed by 1-way ANOVA. Survival probabilities were plotted using the Kaplan-Meier method, and comparison of survival probabilities was performed by the log-rank test. Statistical analyses were performed using GraphPad Prism (version 9). A *P* value of less than 0.05 was considered significant.

### Study approval.

For animal study, all procedures were approved by the Zhejiang University Committee on Use and Care of Animals.

## Author contributions

SZ and Y Sun conceived and planed the study. SZ, XY, YZ, and Y Shen performed experiments. SZ, XY, YZ, Y Shen, XX, and Y Sun analyzed data. SZ, XY, YZ, and Y Sun wrote and revised the manuscript. Y Sun supervised the study. All authors read and approved the manuscript.

## Supplementary Material

Supplemental data

## Version 1 12/22/2022

In-Press Preview

## Version 2 02/15/2023

Electronic publication
