# Supplementary material for: The UBE2C/CDH1/DEPTOR axis is an oncogene and tumor suppressor cascade in lung cancer cells
Source: J Clin Invest. 2023 Feb 15;133(4):e162434. doi: 10.1172/JCI162434 (PMC9927933; doi:10.1172/JCI162434)

Supplemental materials

The UBE2C/CDH1/DEPTOR axis is an oncogene-tumor suppressor cascade in lung cancer cells

Shizhen Zhang,[1,2] Xiahong You,[1,3] Yawen Zheng,[1,3] Yanwen Shen,[1,3] Xiufang Xiong,[1,3] and Yi Sun[1,3,4,5]

[1] Cancer Institute and [2] Department of Breast Surgery and Oncology, Key Laboratory of Cancer Prevention and Intervention, Ministry of Education, the Second Affiliated Hospital, and [3] Institute of Translational Medicine, Zhejiang University School of Medicine, Hangzhou 310029, China.

[4] Zhejiang University Cancer Center, Hangzhou 310029, China.

[5] Research Center for Life Science and Human Health, Binjiang Institute of Zhejiang University, Hangzhou 310053, China

Authorship note: SZ, XY and YZ contributed equally to this work.

Address correspondence to: Yi Sun, Institute of Translational Medicine, Zhejiang University School of Medicine, Hangzhou 310029, China. E-mail: yisun@zju.edu.cn. Phone: 86.15257195968.

Conflict of Interest: The authors claim no conflict of interest.

Running title: UBE2C-APC/C^{CDH1} promotes DEPTOR ubiquitylation

Key Words: UBE2C, DEPTOR, CDH1, APC/C, lung cancer, and ubiquitylation

Supplemental Figures:

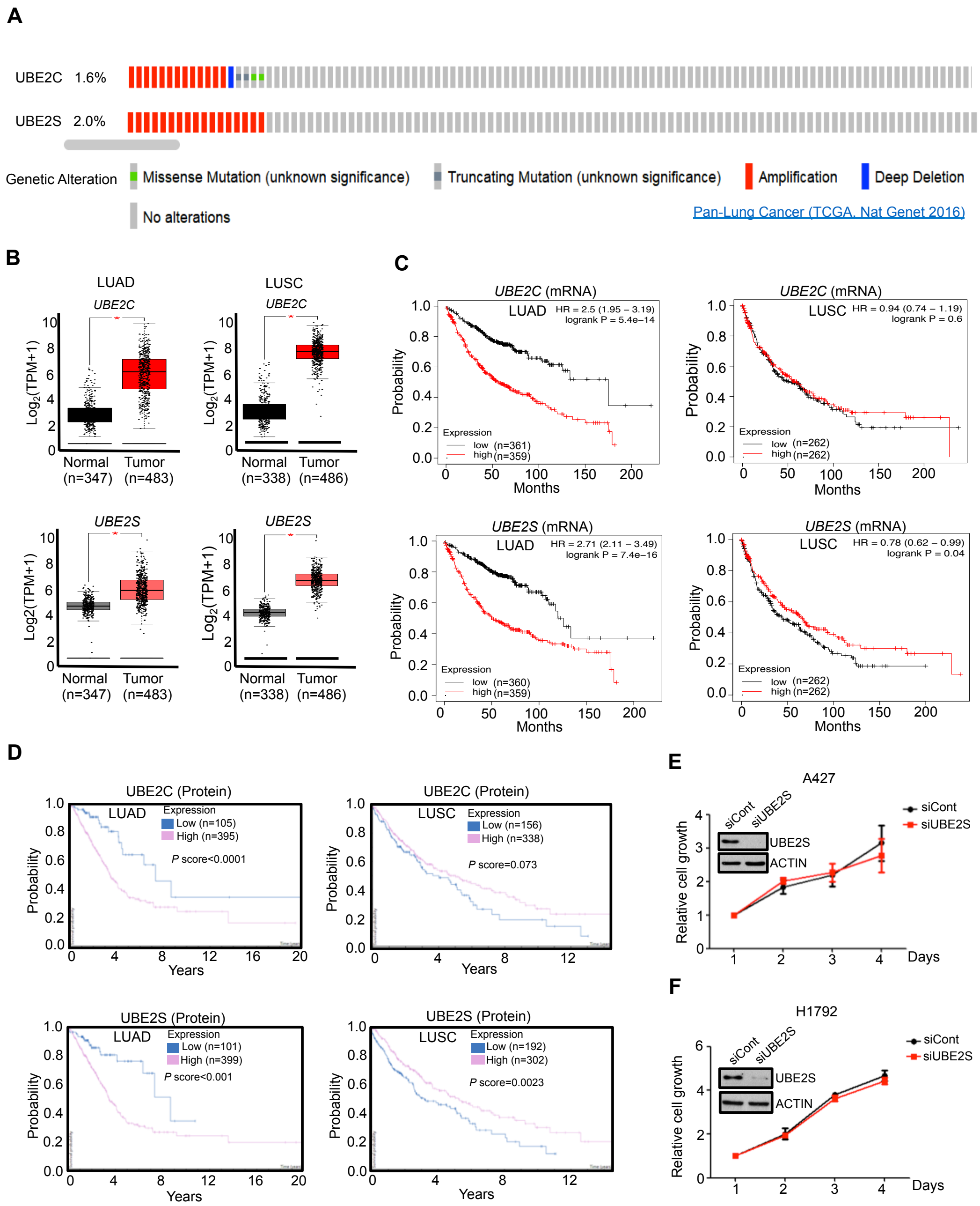

Supplemental Figure 1. Overexpression of UBE2C and UBE2S is associated with worse survival of LUAD patients, while only *UBE2C* knockdown inhibits growth of lung cancer cells

A. The *UBE2C and UBE2S* gene alterations in lung cancer samples analyzed by the cBioPortal database. **B**. Differential expression of *UBE2C* and *UBE2S* between lung cancer (LUAD/LUSC) and normal lung tissues, analyzed by the platform of GEPIA. **C**. Association of *UBE2C/UBE2S* mRNA levels with the survival probability in patients in lung cancer (LUAD/LUSC), analyzed by the Kaplan-Meier Plotter database. **D**. Association of UBE2C/UBE2S protein levels with the survival probability in patients in lung cancer (LUAD/LUSC), analyzed by the Human Protein Atlas. **E-F.** A427 (**E**) and H1792 (**F**) cells were transfected with siRNA targeting *UBE2S* or control siRNA (siCont) for 48 h; then cells were seeded in 96-well plates in triplicate and subjected to a CCK-8 cell proliferation assay or IB (inset). The mean ± SEM are shown from three independent experiments. LUAD: lung adenocarcinoma, LUSC: lung squamous cell carcinoma.

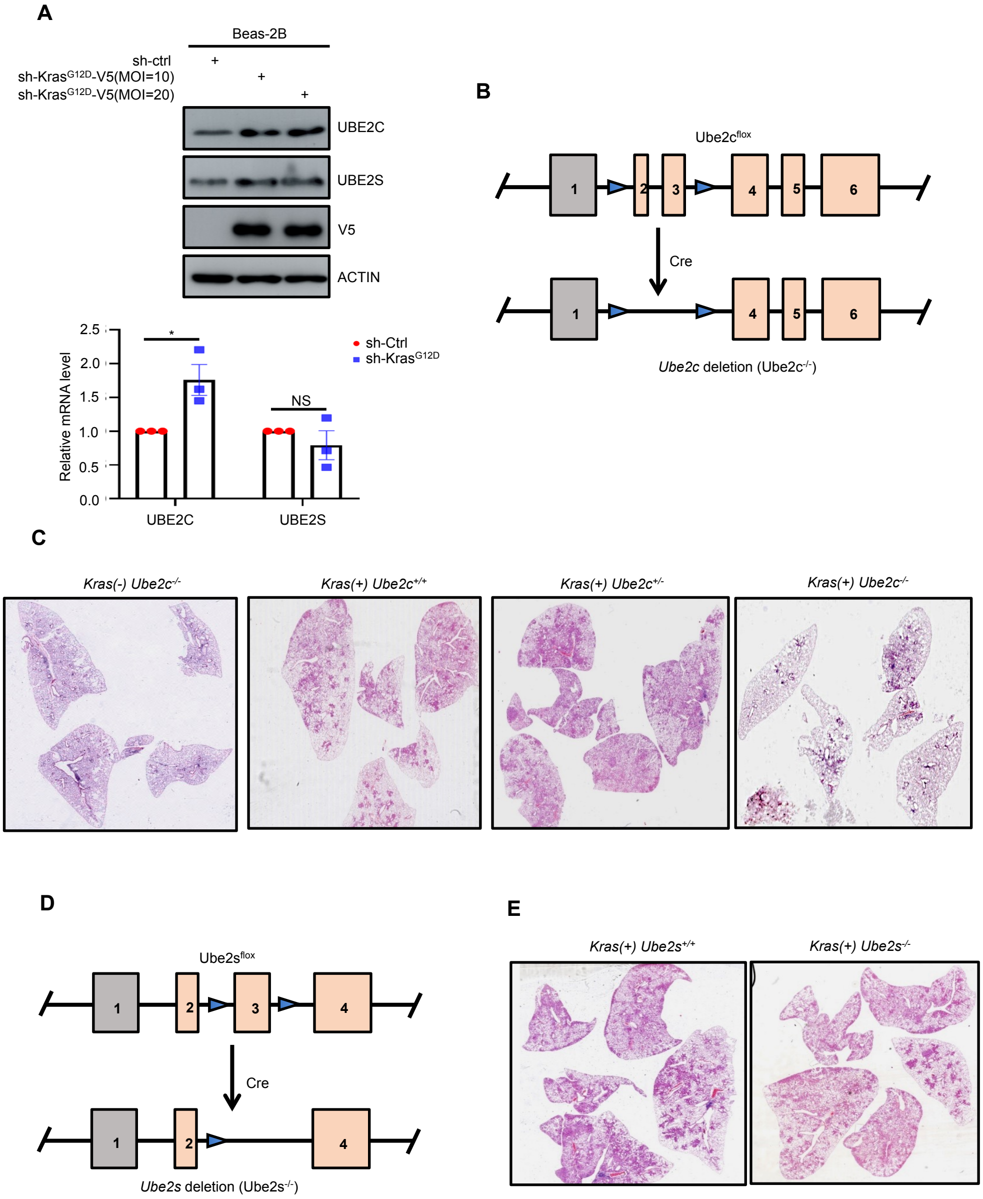
A
Beas-2B
sh-ctrl
sh-KrasG12D-V5(MOI=10)
sh-KrasG12D-V5(MOI=20)
UBE2C
UBE2S
V5
ACTIN
Relative mRNA level
2.5
2.0
1.5
1.0
0.5
0.0
*
NS
sh-Ctrl
sh-KrasG12D
UBE2C
UBE2S
B
Ube2cflox
Cre
Ube2c deletion (Ube2c-/-)
C
Kras(-) Ube2c-/-
Kras(+) Ube2c+/+
Kras(+) Ube2c+/-
Kras(+) Ube2c-/-
D
Ube2sflox
Cre
Ube2s deletion (Ube2s-/-)
E
Kras(+) Ube2s+/+
Kras(+) Ube2s-/-

Supplemental Figure 2. The diagram of conditional knockout strategy

A. Beas-2B cells were transfected with sh-Kras^{G12D}-V5, followed by IB with indicated antibodies (top), or by RT-qPCR analysis (bottom). **B**. The Cas9/RNA system technology was used to target exons 2 and 3 in *Ube2c* with flanking loxP sites for construction of *Ube2c*$^{fl/fl}$ mice. **C**. The lung tissues of five lobes were isolated from mice with indicated genotypes, fixed, sectioned and subjected to H&E staining. **D.** The Cas9/RNA system technology was used to target exons 3 in *Ube2s* with flanking loxP sites for construction of *Ube2s*$^{fl/fl}$ mice. **E.** The lung tissues of five lobes from mice with indicated genotypes were fixed, sectioned and subjected to H&E staining. $P < 0.05$ (*), by 2-tailed Student's *t* test (**A**).

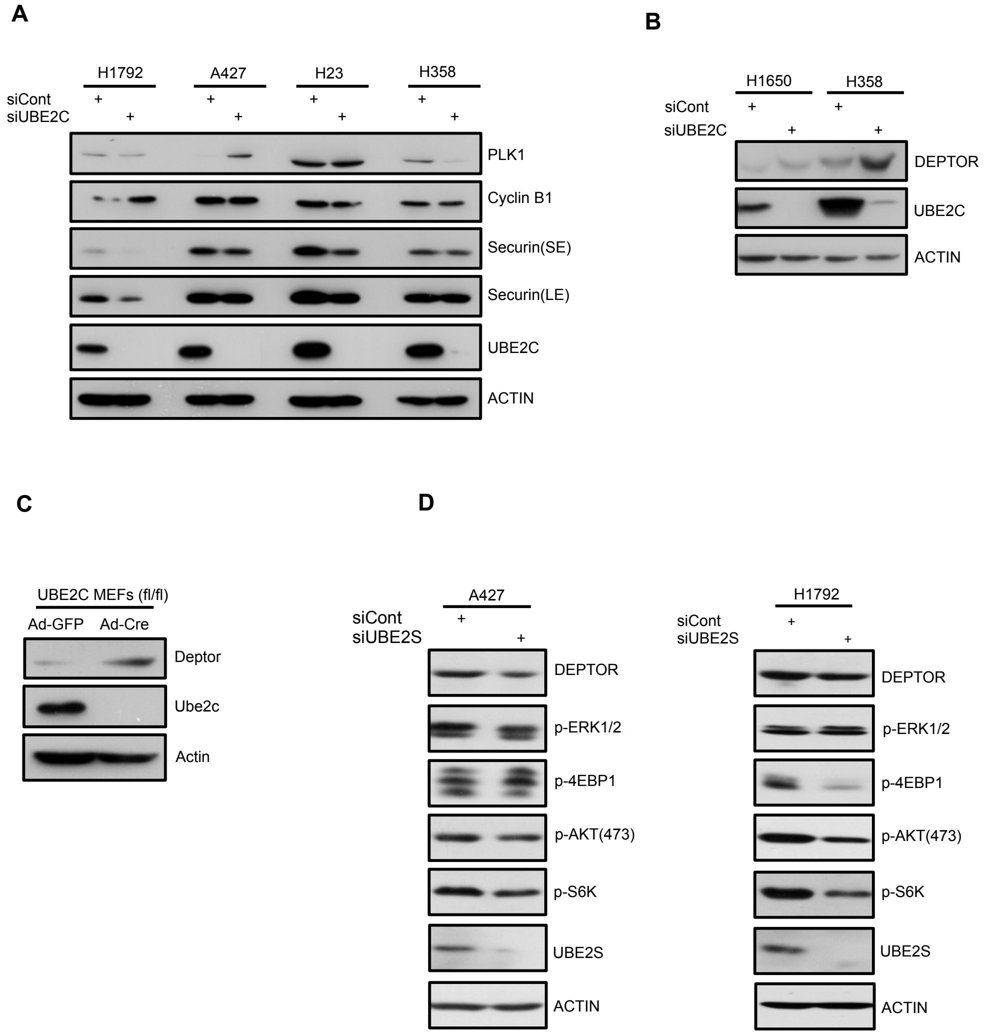

Supplemental Figure 3. UBE2C differentially regulates mTOR signaling and DEPTOR levels

A. H1792, A427, H23 and H358 cells were transfected with siRNA targeting UBE2C or siCont for 48 h, followed by IB with indicated antibodies. **B**. H1650 and H358 cells were transfected with siRNA targeting UBE2C or siCont for 48 h, followed by IB with indicated antibodies. **C.** MEF cells were infected with ad-GFP or ad-Cre for 72 h and subjected to IB with indicated antibodies. **D**. H1792 and A427 cells were transfected with siRNA targeting UBE2CS or siCont for 48 h, followed by IB with indicated antibodies. SE: short exposure, LE: long exposure.

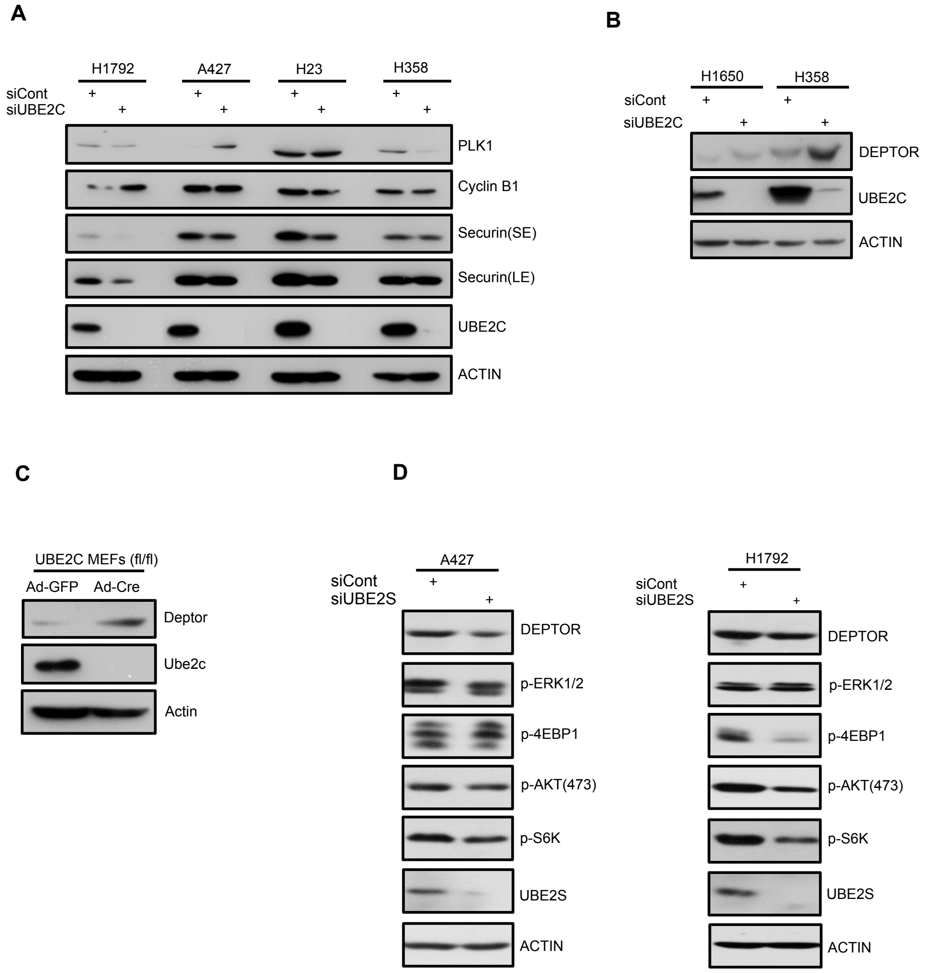

Supplemental Figure 4. DEPTOR is a cell-cycle regulatory protein, controlled by the UBE2C/CDH1 axis

A. H1792 cells were infected with indicated siRNAs followed by IB with the indicated Abs (top), or by RT-qPCR analysis (bottom). **B**. H1792 and H358 cells were infected with indicated siRNAs followed by IB with the indicated antibodies. **C-D**. H1792 cells were transfected with siRNA targeting UBE2C (C) or CDH1 (D), along with siCont for 24 h, then cells were synchronized at the G1/S phase by thymidine block and released for indicated time points. Cells were harvested for IB analysis with indicated antibodies. **E**. HeLa cells were transfected with siRNA targeting β-TrCP or siCont for 24 h, then cells were released from G1/S phase arrested by double thymidine block into normal cell cycle at indicated time, and subjected to IB analysis with indicated antibodies.

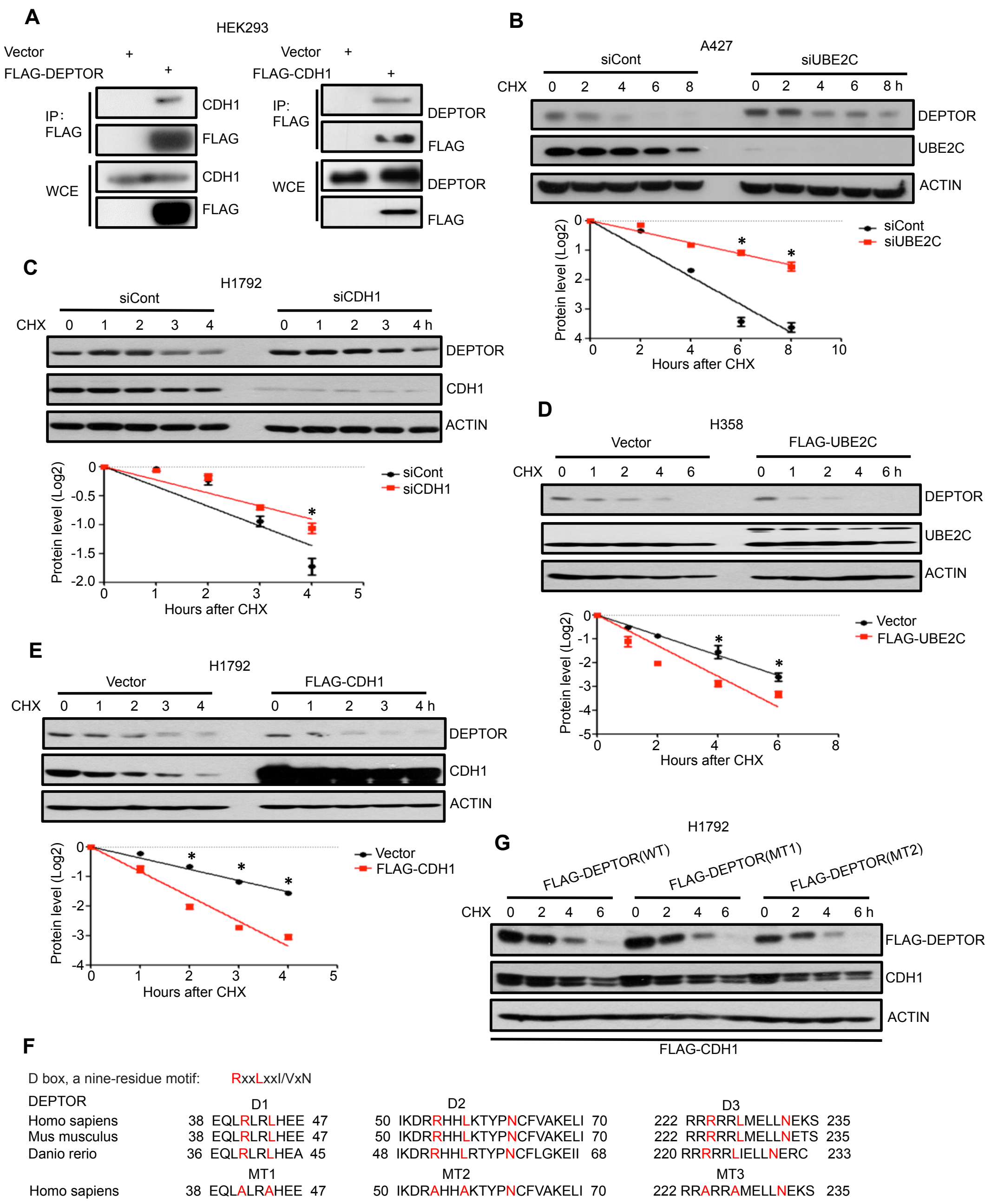
A
HEK293
Vector
FLAG-DEPTOR
IP: FLAG
WCE
CDH1
FLAG
FLAG-CDH1
DEPTOR
B
A427
siCont
siUBE2C
CHX 0 2 4 6 8 0 2 4 6 8 h
DEPTOR
UBE2C
ACTIN
Protein level (Log2)
Hours after CHX
siCont
siUBE2C
C
H1792
siCont
siCDH1
CHX 0 1 2 3 4 0 1 2 3 4 h
DEPTOR
CDH1
ACTIN
Protein level (Log2)
Hours after CHX
siCont
siCDH1
D
H358
Vector
FLAG-UBE2C
CHX 0 1 2 4 6 0 1 2 4 6 h
DEPTOR
UBE2C
ACTIN
Protein level (Log2)
Hours after CHX
Vector
FLAG-UBE2C
E
H1792
Vector
FLAG-CDH1
CHX 0 1 2 3 4 0 1 2 3 4 h
DEPTOR
CDH1
ACTIN
Protein level (Log2)
Hours after CHX
Vector
FLAG-CDH1
G
H1792
FLAG-DEPTOR(WT)
FLAG-DEPTOR(MT1)
FLAG-DEPTOR(MT2)
CHX 0 2 4 6 0 2 4 6 0 2 4 6 h
FLAG-DEPTOR
CDH1
ACTIN
FLAG-CDH1
F
D box, a nine-residue motif: RxxLxxI/VxN
DEPTOR D1 D2 D3
Homo sapiens 38 EQLRLRLHEE 47 50 IKDRRHHLKTYPNCFVAKELI 70 222 RRRRRLMELLNEKS 235
Mus musculus 38 EQLRLRLHEE 47 50 IKDRRHHLKTYPNCFVAKELI 70 222 RRRRRLMELLNETS 235
Danio rerio 36 EQLRLRLHEA 45 48 IKDRRHHLRTYPNCFLGKEII 68 220 RRRRRLIELLNERC 233
MT1 MT2 MT3
Homo sapiens 38 EQLALRAHEE 47 50 IKDRAHHAKTYPNCFVAKELI 70 222 RRARRAMELLNEKS 235

Supplemental Figure 5. DEPTOR is a new substrate of UBE2C-APC/C^{CDH1} E2-E3 complex

A. HEK293 cells were transfected with plasmids expressing FLAG-tagged DEPTOR or FLAG-tagged CDH1 or vector control for 48 h with last 8-h treatment of 10 mM MG132 before harvesting. Cell lysates were prepared for immunoprecipitation (IP) with immunoglobulin G (IgG) control or antibody against FLAG, followed by IB with indicated antibodies. WCE, whole-cell extract. **B-C**. A427 (B) and H1792 (C) cells were transfected with indicated siRNAs for 48 h, cells were then treated with cycloheximide (CHX) for the indicated time periods and harvested for IB (top). The band density was quantified using Image J software, and the decay curves were drawn (bottom). **D-E**. H358 (D) and H1792 (E) cells were transfected with indicated plasmids for 48 h, cells were then treated with CHX for the indicated time periods and harvested for IB (top). The decay curves were drawn (bottom). **F**. Evolutionary conservation of the D-box motif on DEPTOR, and the schematic representation of the D box mutants (DEPTOR-MT1, MT2 and MT3) generated. **G**. Cells were then treated with CHX for the indicated time periods and harvested for IB. $P < 0.05$ (*) and $P < 0.01$ (**), by 1-way ANOVA test (B-E).

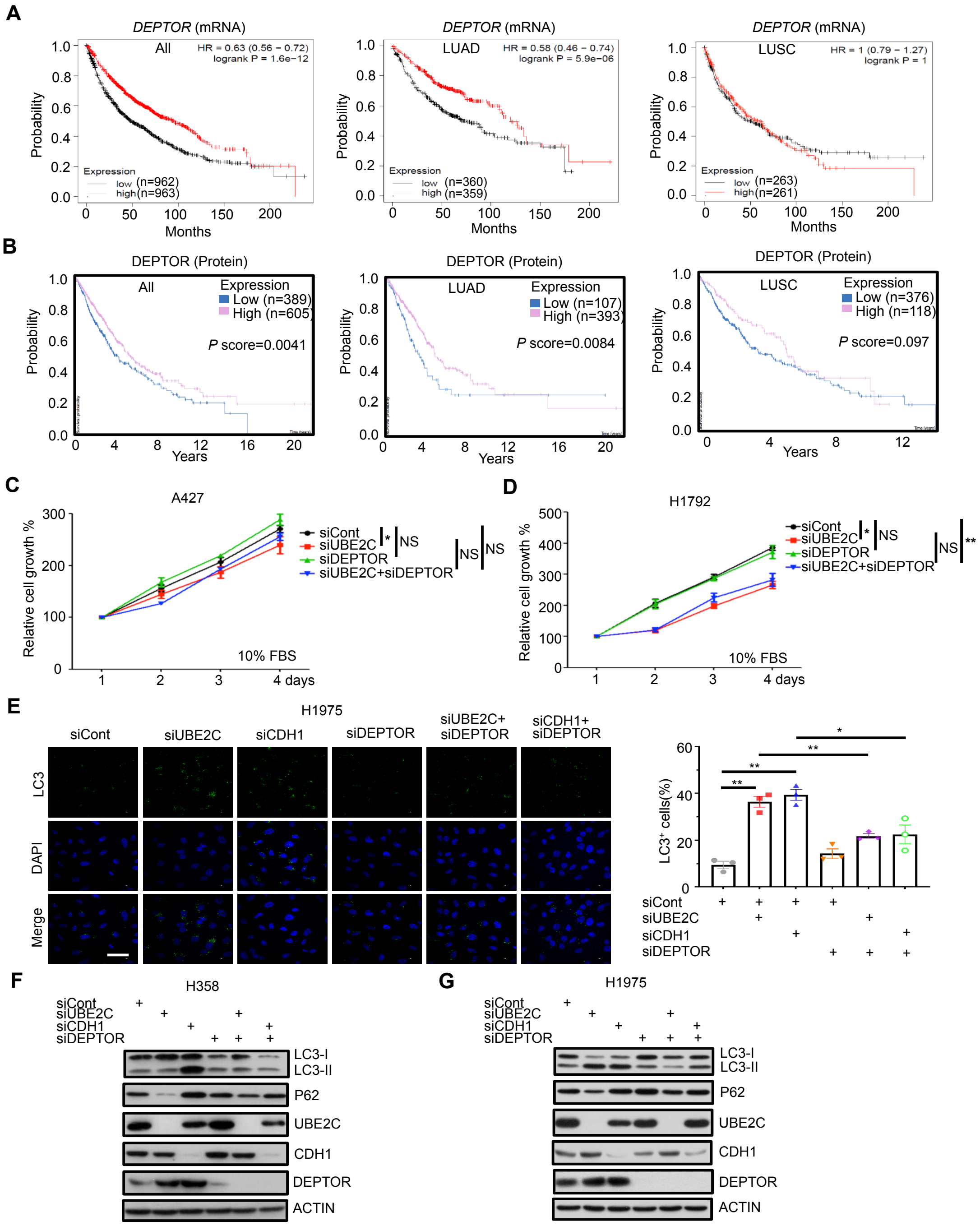
A
DEPTOR (mRNA)
All
HR = 0.63 (0.56 − 0.72)
logrank P = 1.6e−12
Probability
Expression
low (n=962)
high (n=963)
Months
LUAD
HR = 0.58 (0.46 − 0.74)
logrank P = 5.9e−06
low (n=360)
high (n=359)
LUSC
HR = 1 (0.79 − 1.27)
logrank P = 1
low (n=263)
high (n=261)
B
DEPTOR (Protein)
Expression
Low (n=389)
High (n=605)
P score=0.0041
Years
Low (n=107)
High (n=393)
P score=0.0084
Low (n=376)
High (n=118)
P score=0.097
C
A427
Relative cell growth %
siCont
siUBE2C
siDEPTOR
siUBE2C+siDEPTOR
NS
10% FBS
4 days
D
H1792
E
H1975
siCont
siUBE2C
siCDH1
siDEPTOR
siUBE2C+ siDEPTOR
siCDH1+ siDEPTOR
LC3
DAPI
Merge
LC3+ cells(%)
F
H358
LC3-I
LC3-II
P62
UBE2C
CDH1
DEPTOR
ACTIN
G
H1975

Supplemental Figure 6. *DEPTOR* knockdown rescues the phenotypes induced by *UBE2C* knockdown in lung cancer cells

A. The prognostic values of *DEPTOR* mRNA expression in lung cancer (LUAD/LUSC) analyzed by the Kaplan-Meier Plotter database. **B**. The prognostic values of DEPTOR protein expression in lung cancer (LUAD/LUSC) analyzed by the Human Protein Altas. **C-D**. A427 (C) and H1792 (D) cells were transfected with indicated siRNAs for 24 h; then cells were seeded in 96-well plates with medium containing 10% FBS in triplicate and subjected to a CCK-8 cell proliferation assay. **E**. H1975 cell were transfected with indicated siRNAs for 48 h, stained with indicated Abs, followed by photography under fluorescent microscope (left). Data are the mean ± SEM of three independent experiments (right). **F-G**. H358 (**F**) and H1975 (**G**) cells were transfected with indicated siRNAs for 48 h, followed by IB with indicated antibodies. $P < 0.05$ (*) and $P < 0.01$ (**), by 1-way ANOVA test (**C-E**). Scale bars, 50μm.

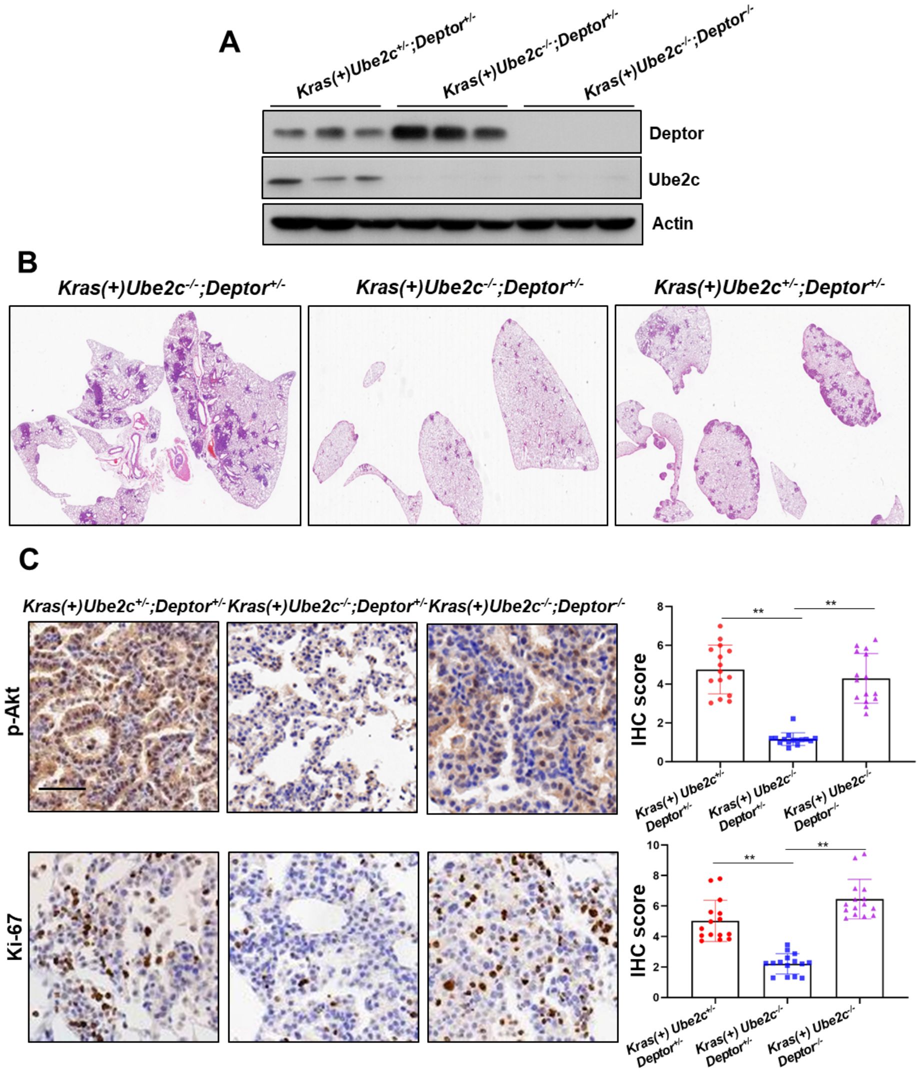

Supplemental Figure 7. *Deptor* KO rescues the phenotypes induced by *Ube2c* knockout *in vivo*

A. Lung tissues isolated from three individual mice with indicated genotypes were milled and lysed with lysis buffer containing complete protease inhibitor cocktail, then subjected to IB with indicated antibodies. **B.** The lung tissues of five lobes were isolated from mice with indicated genotypes, fixed, sectioned and subjected to H&E staining. **C**. The lung tissues were isolated from mice with three indicated genotypes, fixed, sectioned and subjected to immunohistochemical staining with indicated antibodies. The staining quantification was analyzed by the semiquantitative immunoreactivity scoring system as described in the materials and methods section. $P < 0.01$ (**), by 1-way ANOVA test (**C**). Scale bars, 50μm.

Full unedited gel for Figure 1

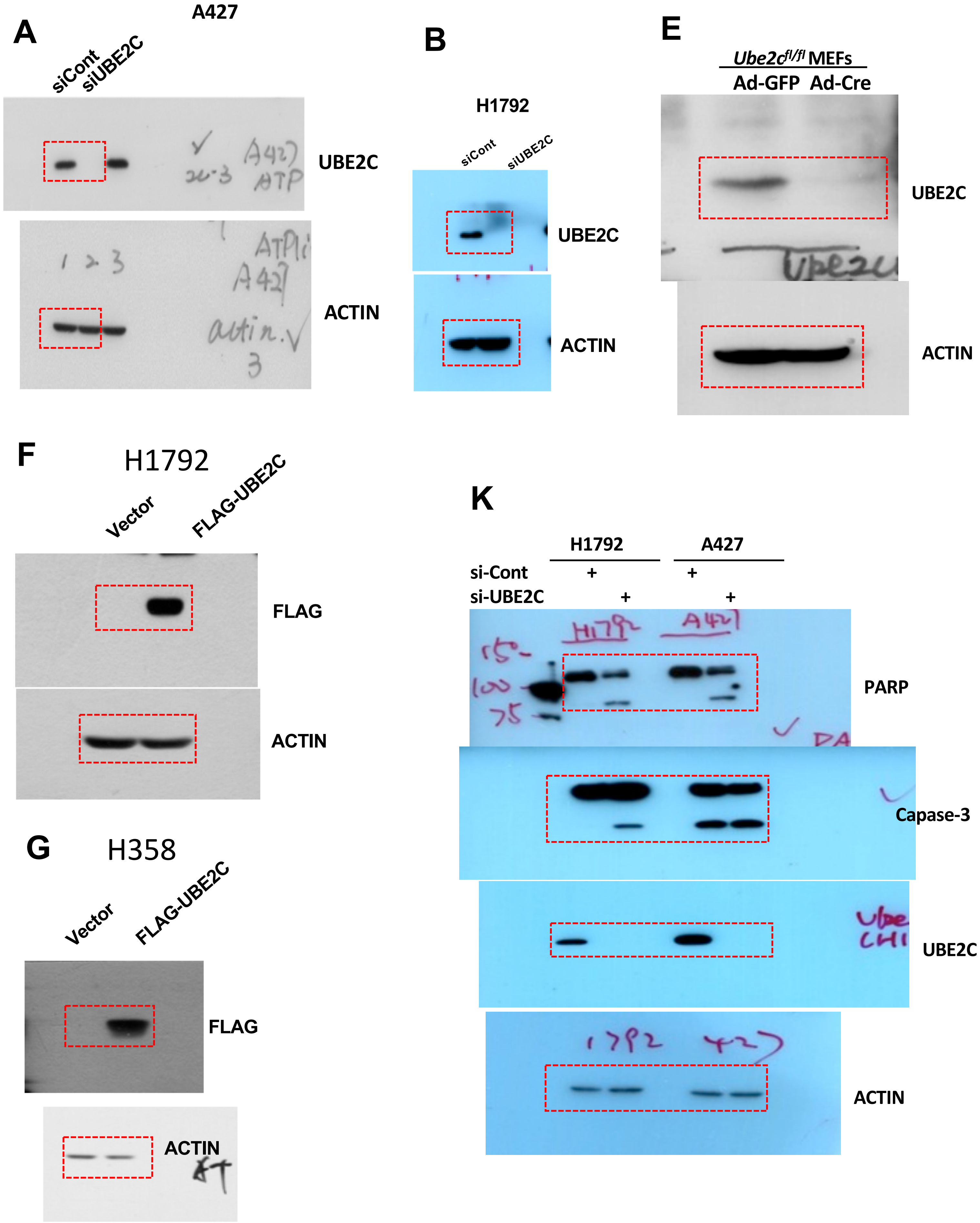

Full unedited gel for Figure 2

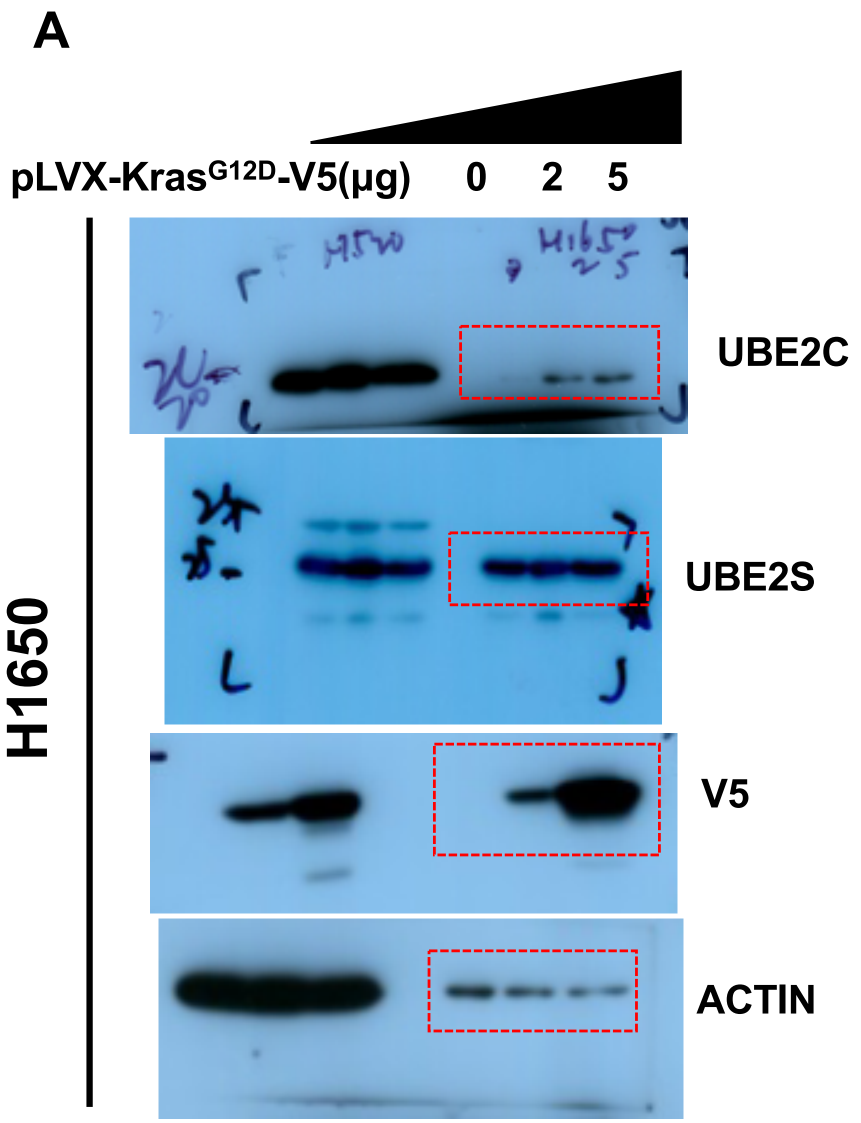

Full unedited gel for Figure 3

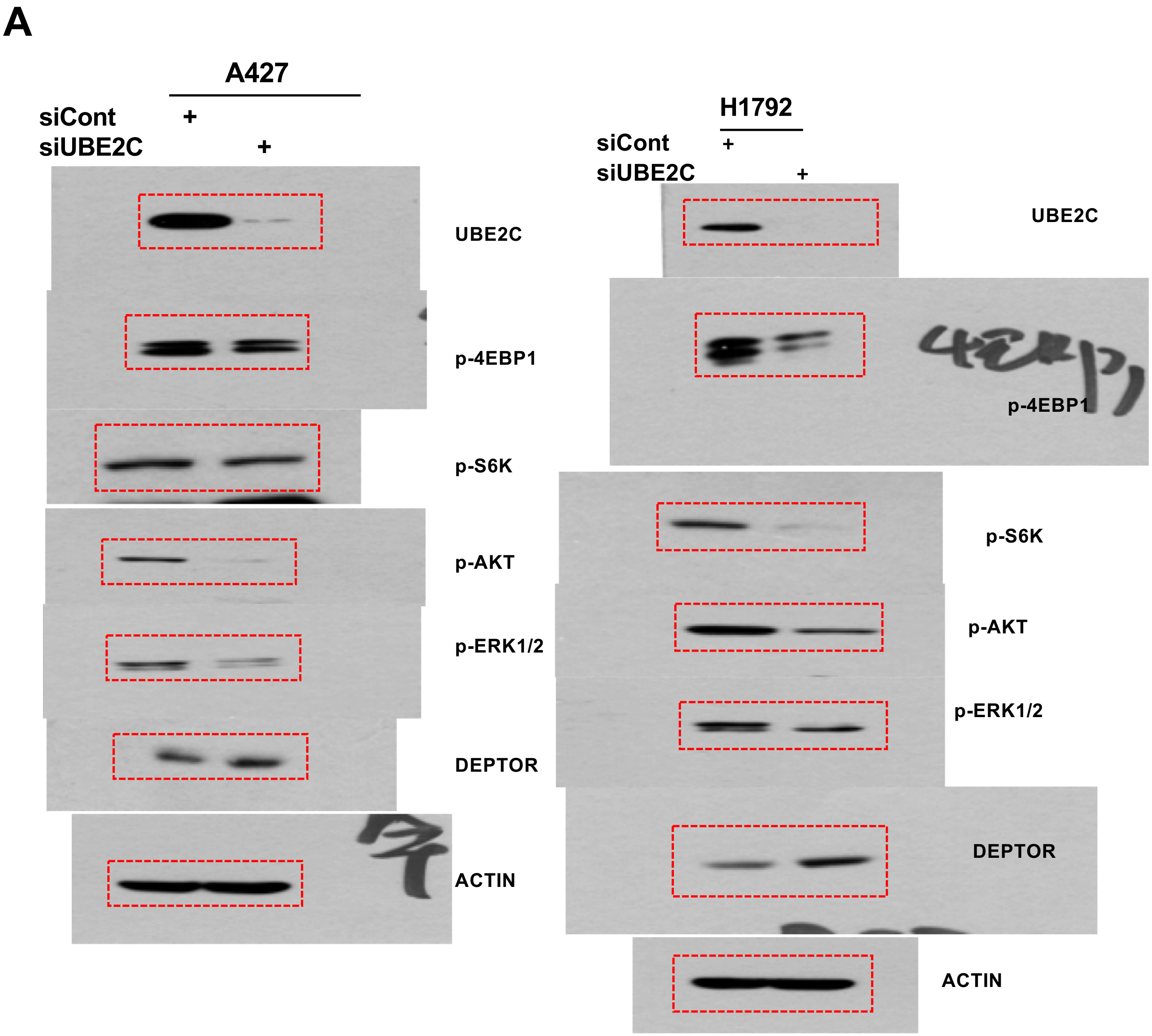

Full unedited gel for Figure 3

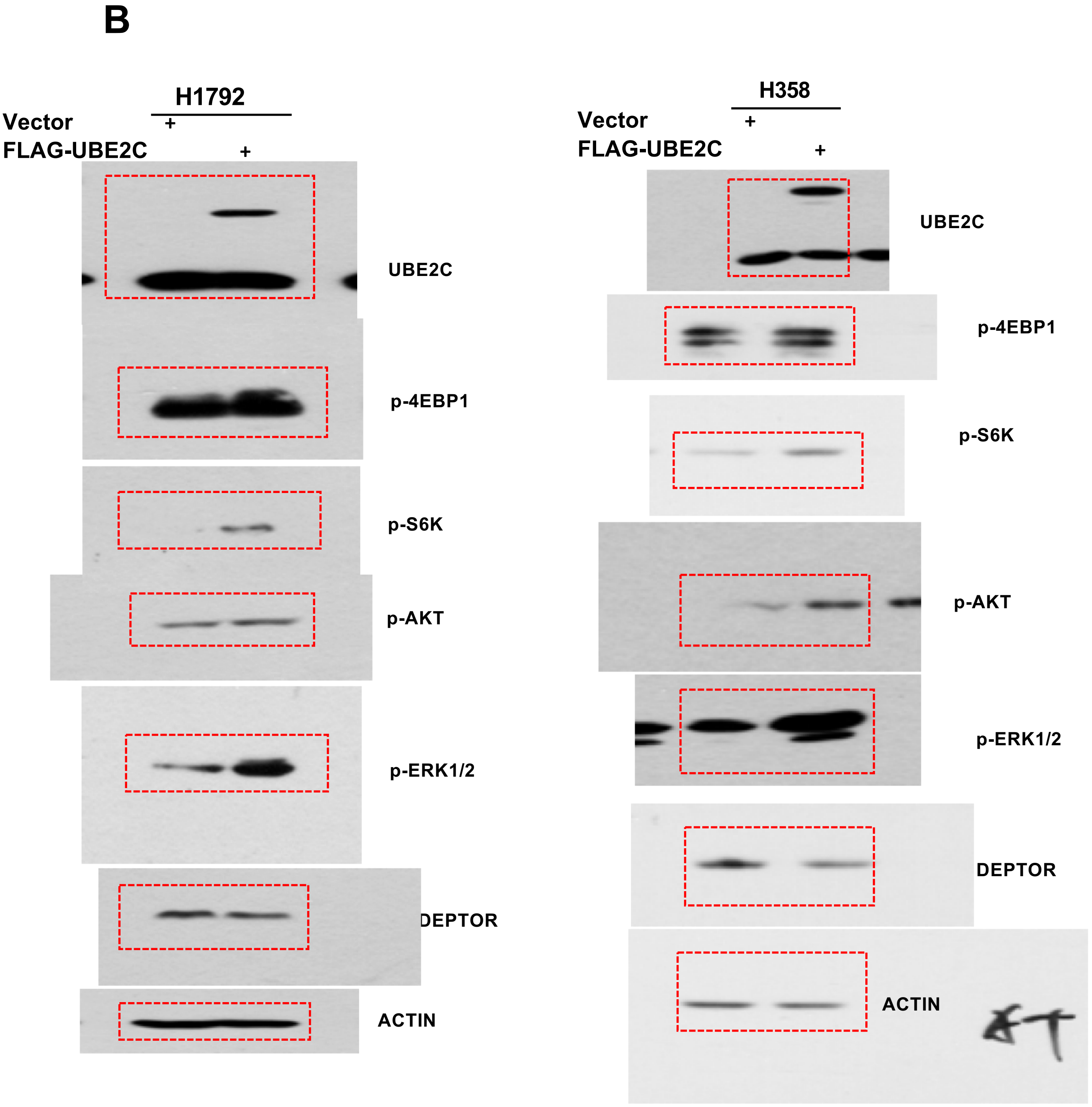

Full unedited gel for Figure 4

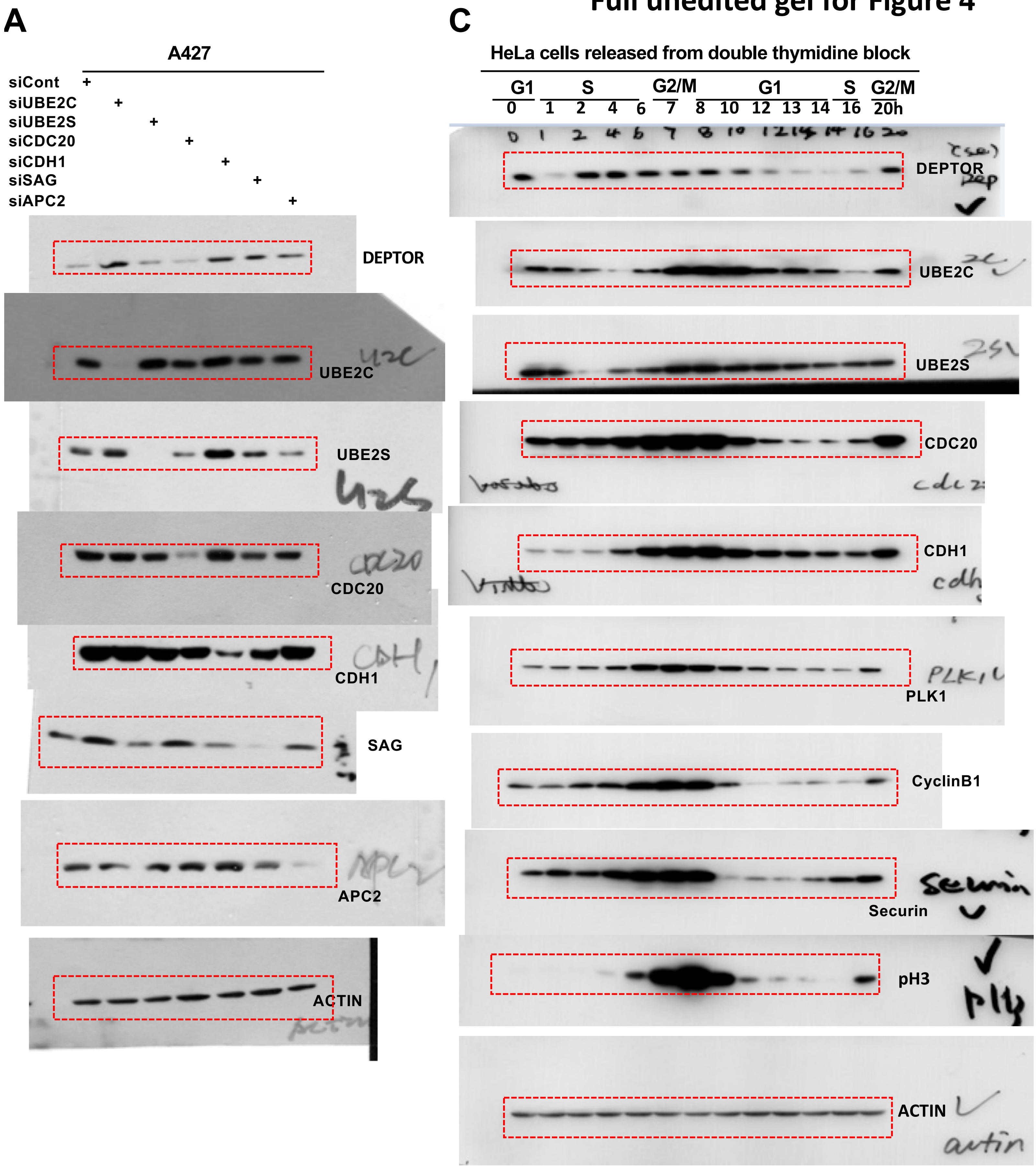

Full unedited gel for Figure 4

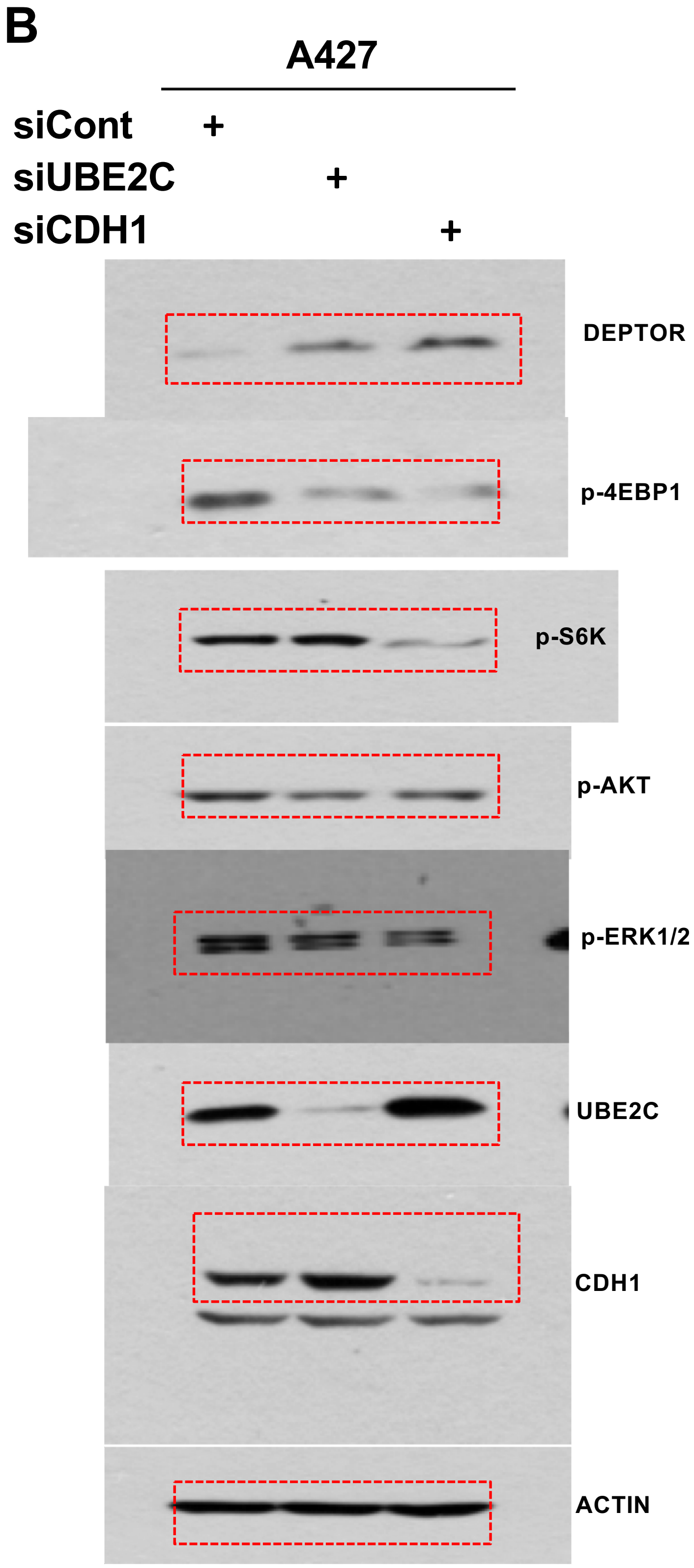

Full unedited gel for Figure 4

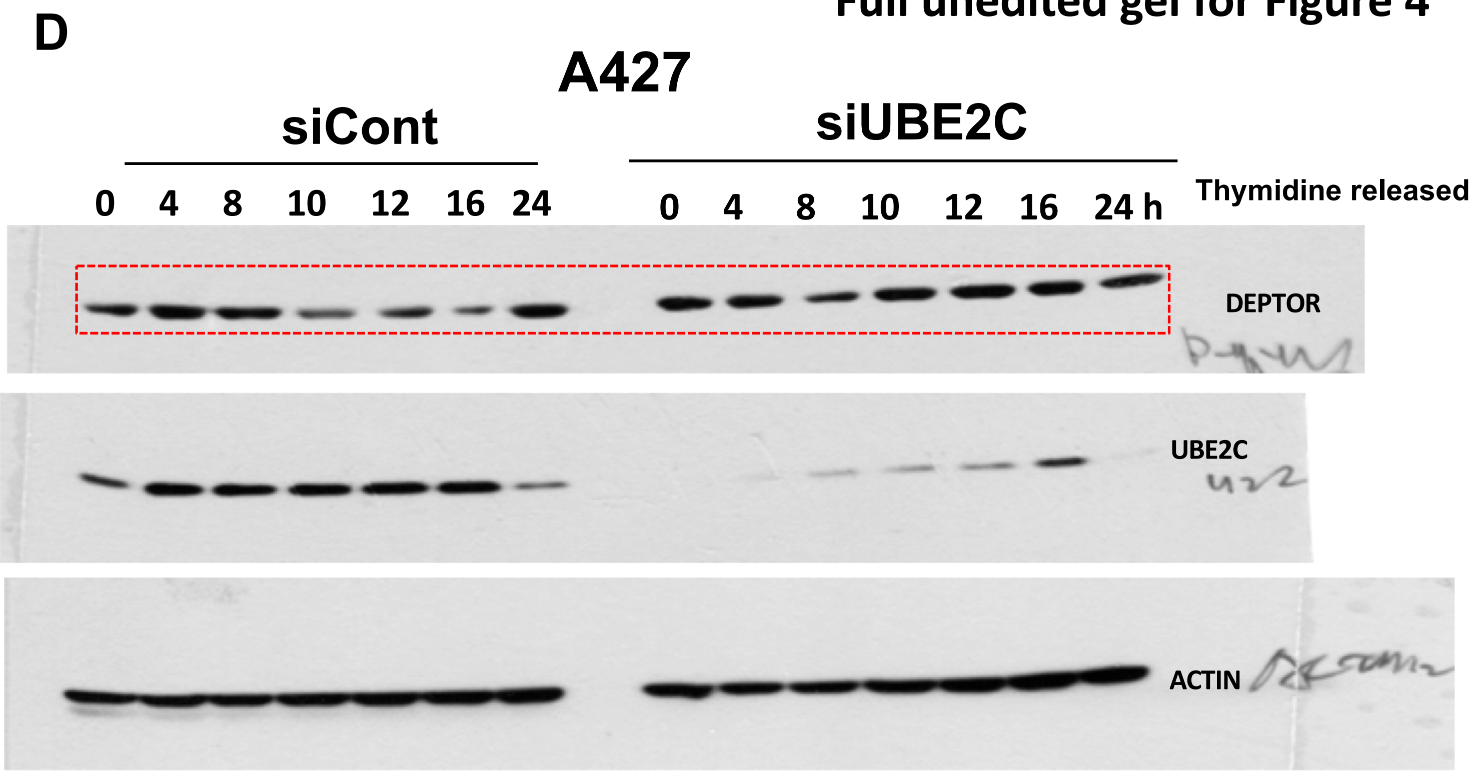

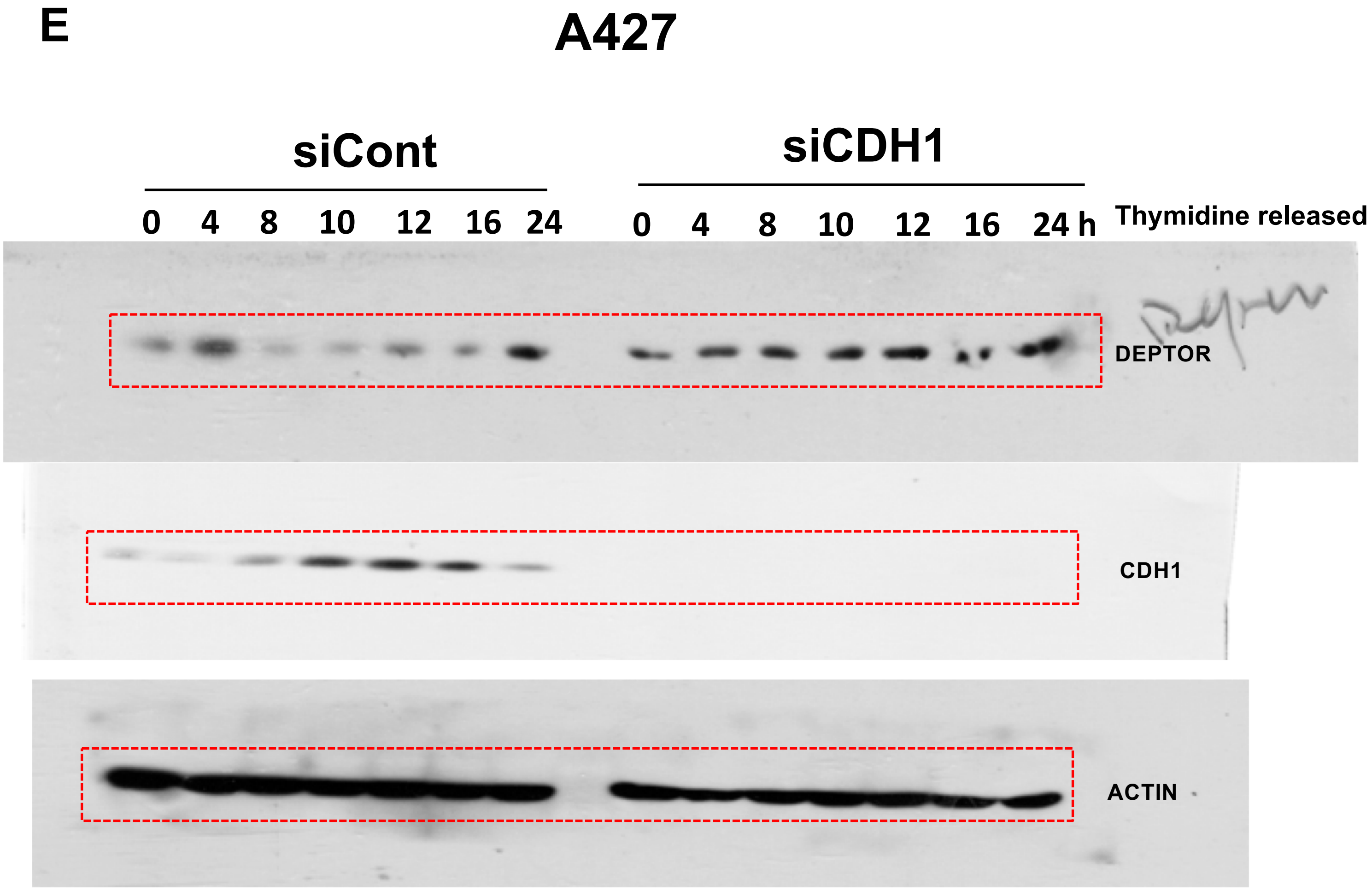

Full unedited gel for Figure 5

A

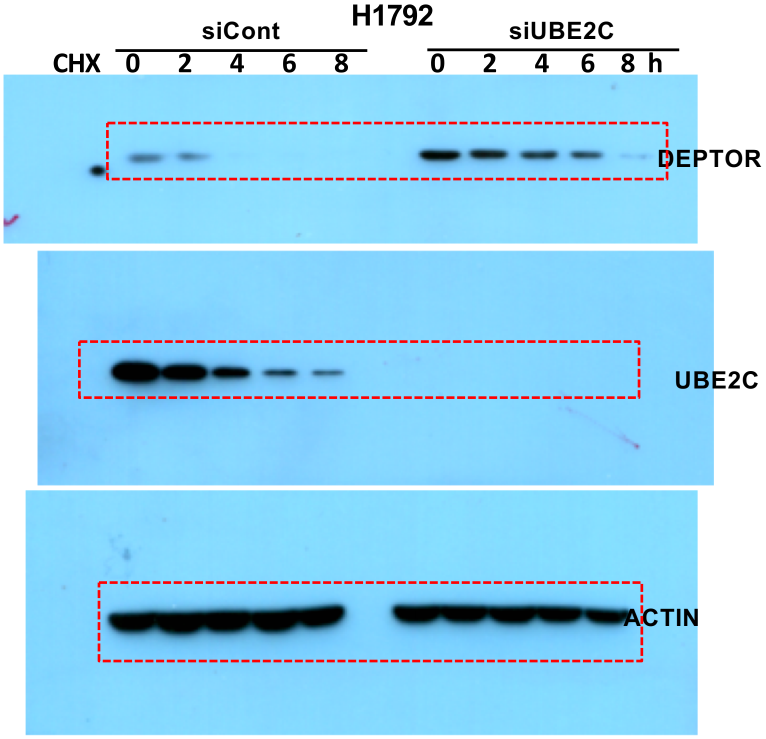

B

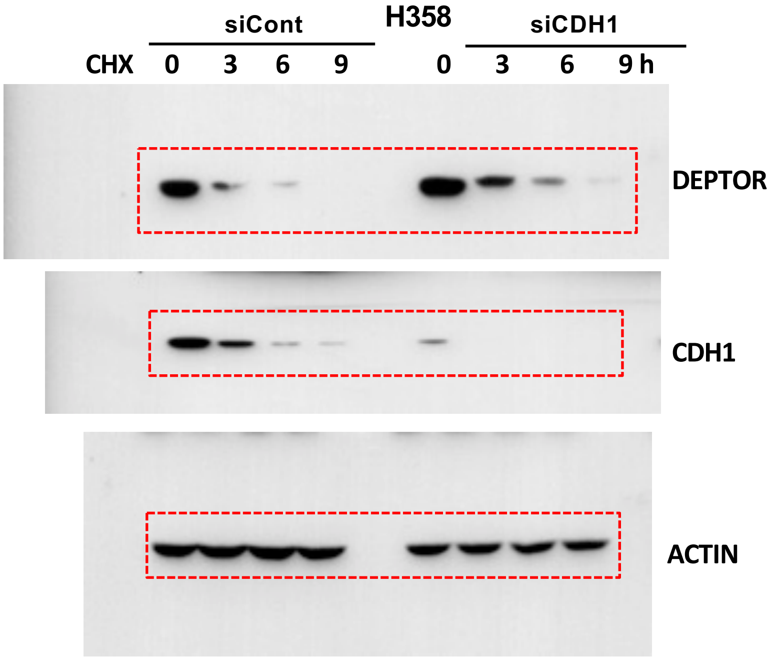

C

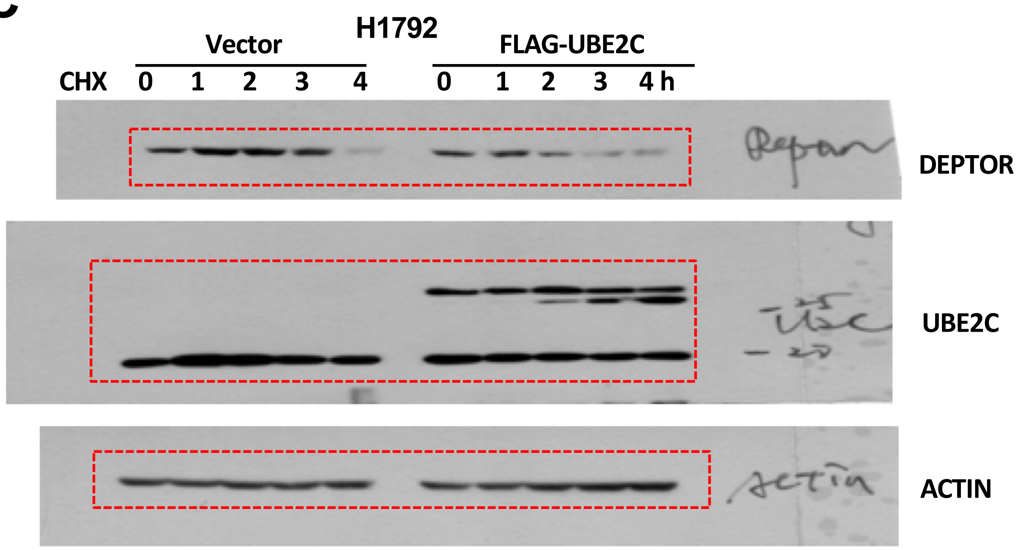

Full unedited gel for Figure 5

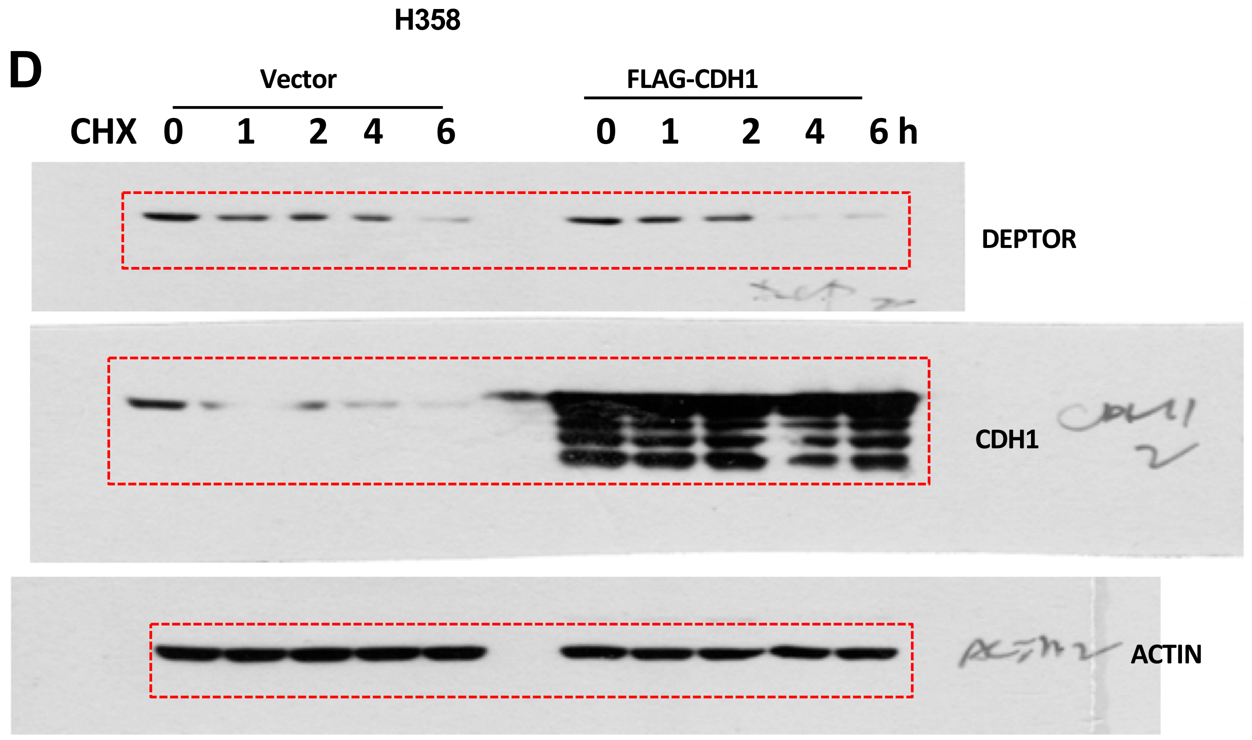

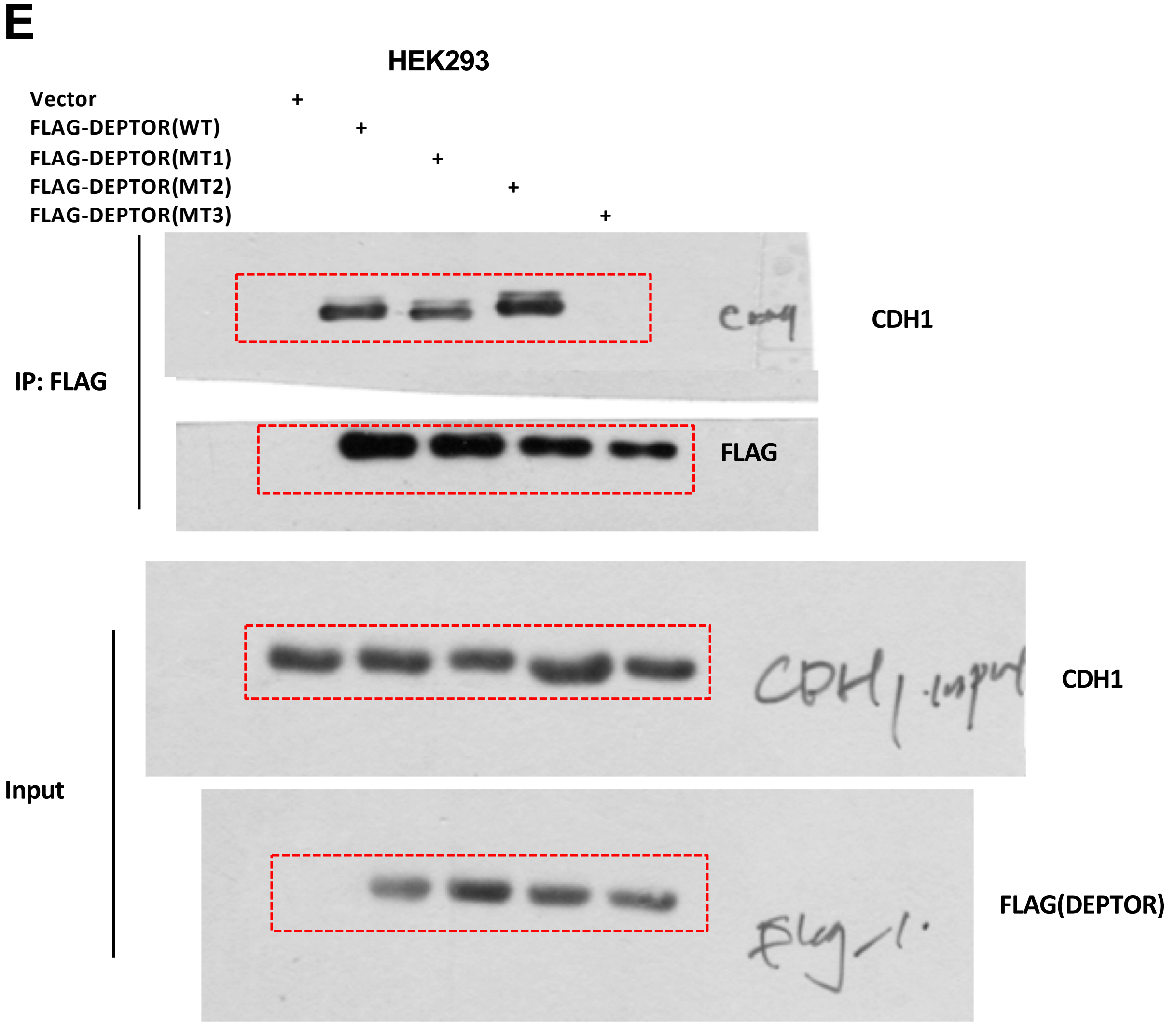

Full unedited gel for Figure 5

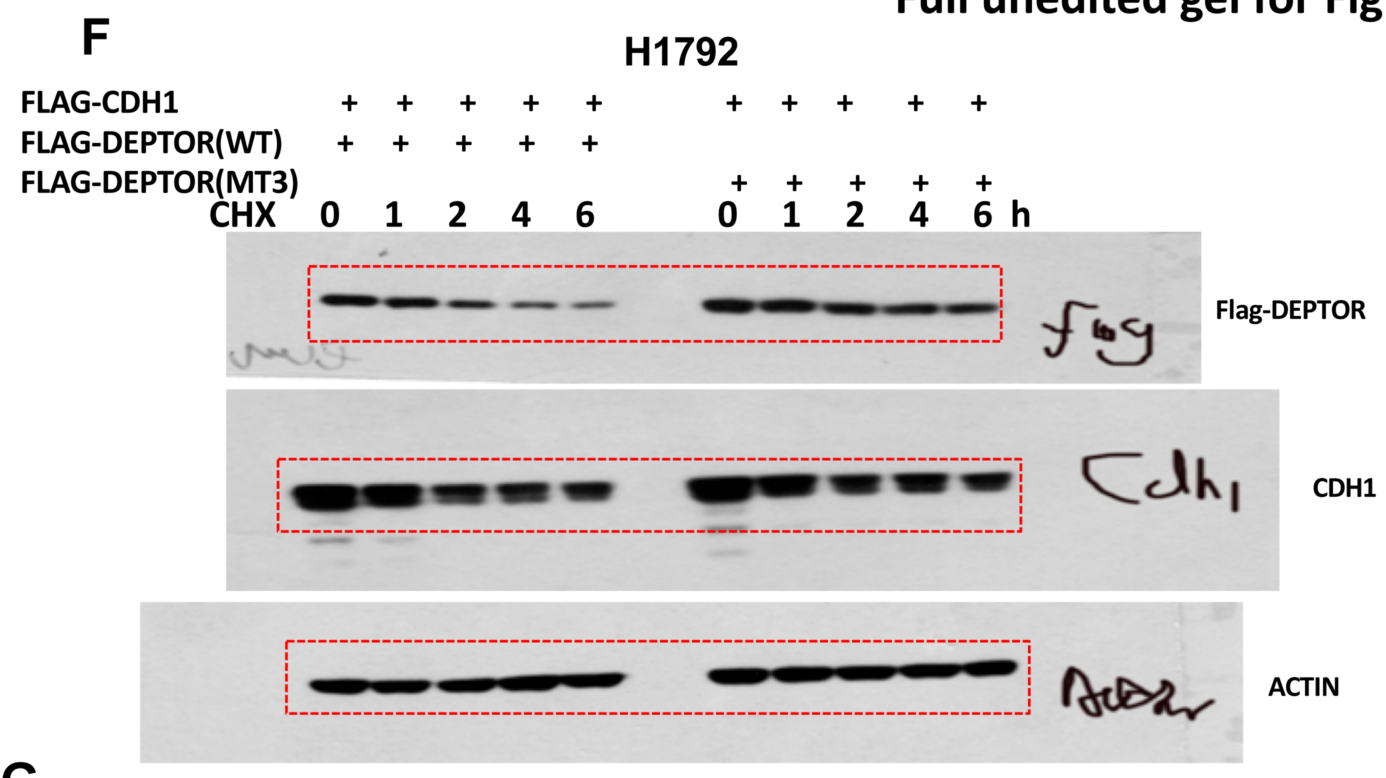

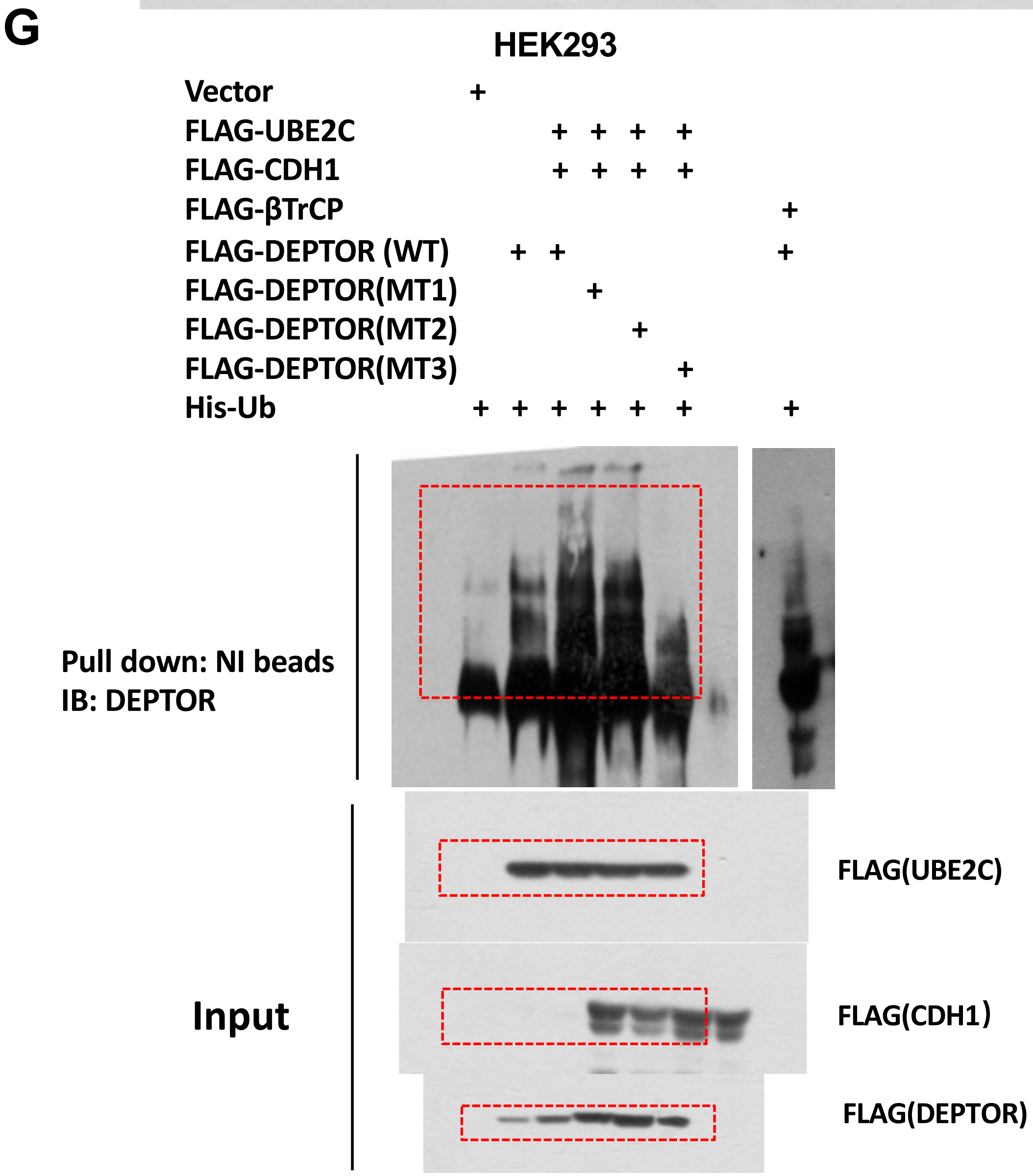

Full unedited gel for Figure 6

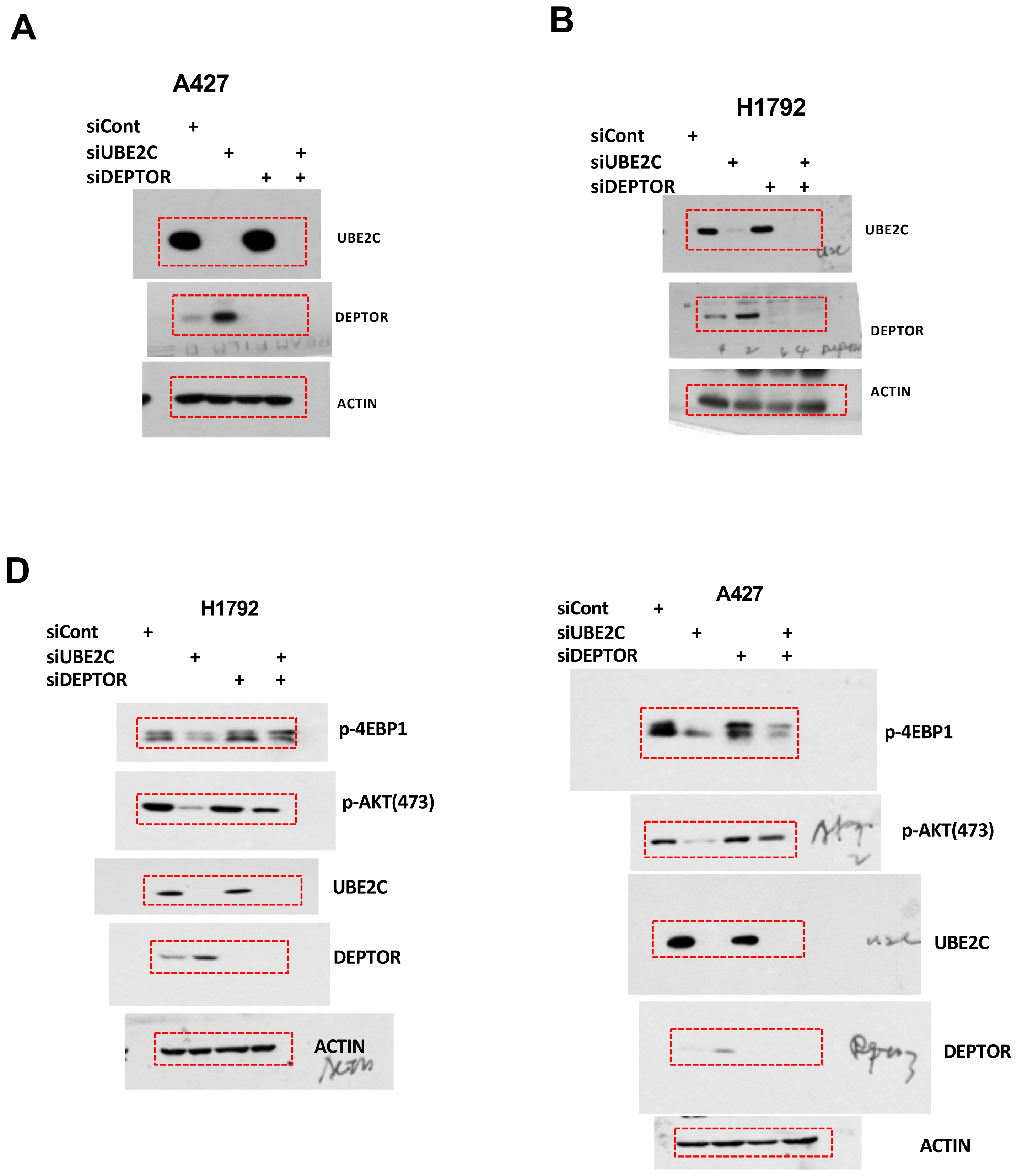

Full unedited gel for Figure S1

E

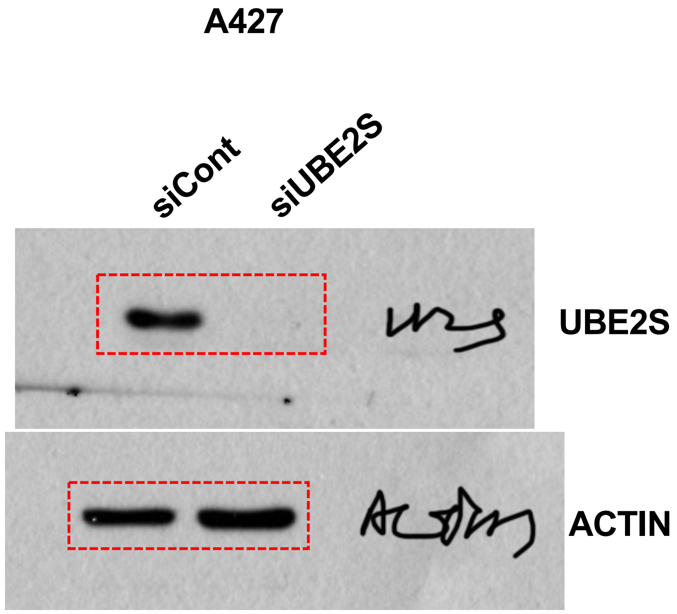

F

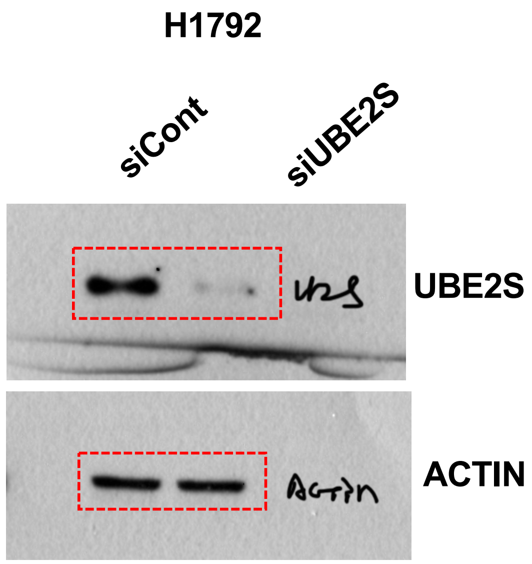

Full unedited gel for Figure S2

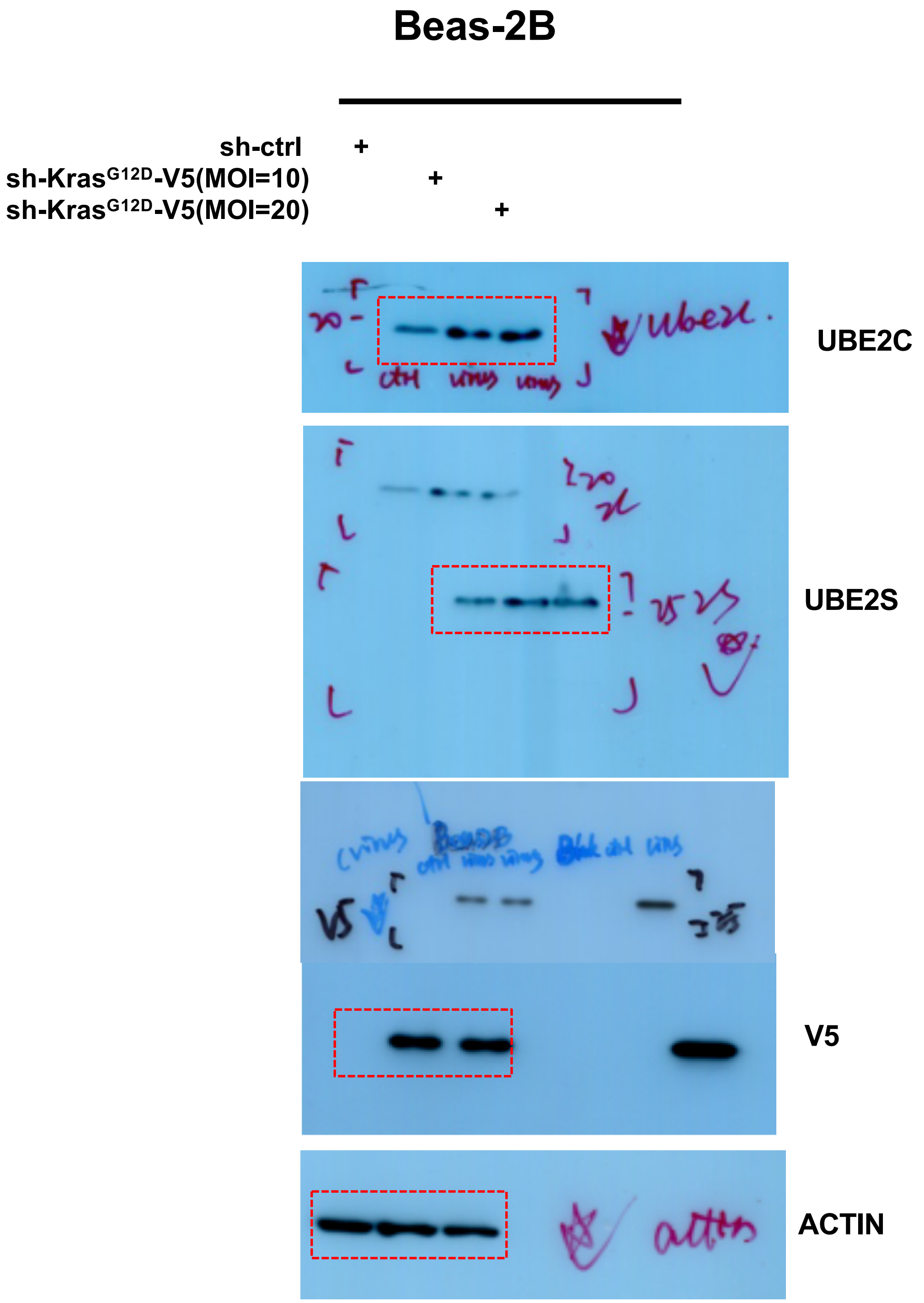

Full unedited gel for Figure S3

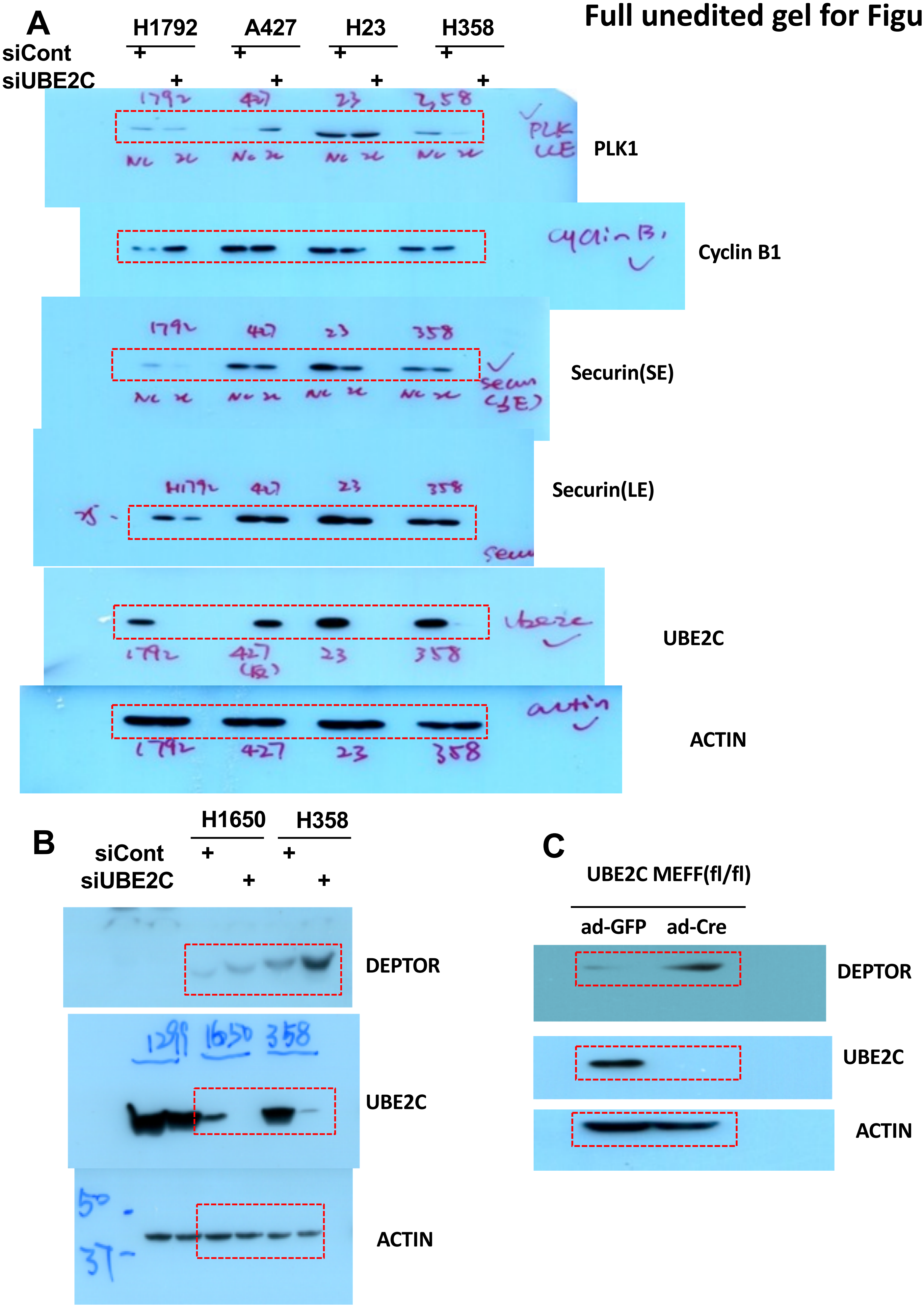

Full unedited gel for Figure S3

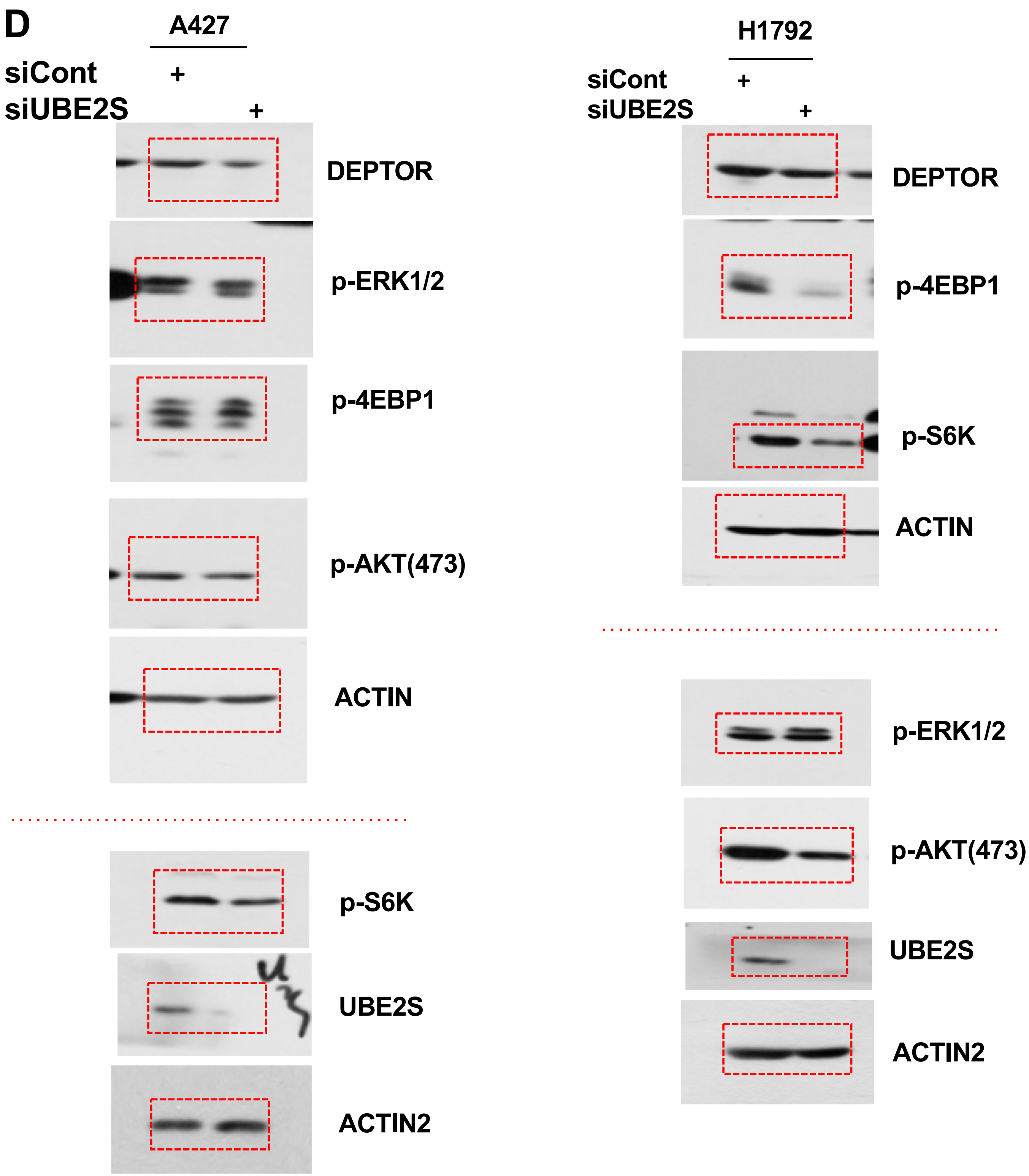

Full unedited gel for Figure S4

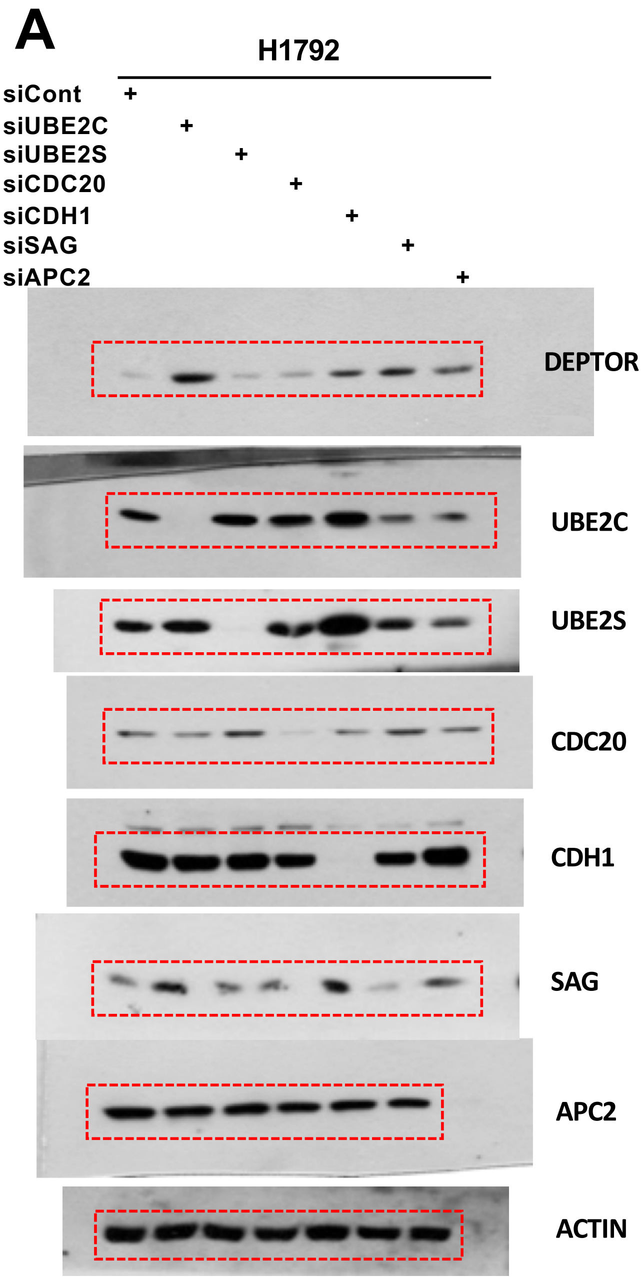

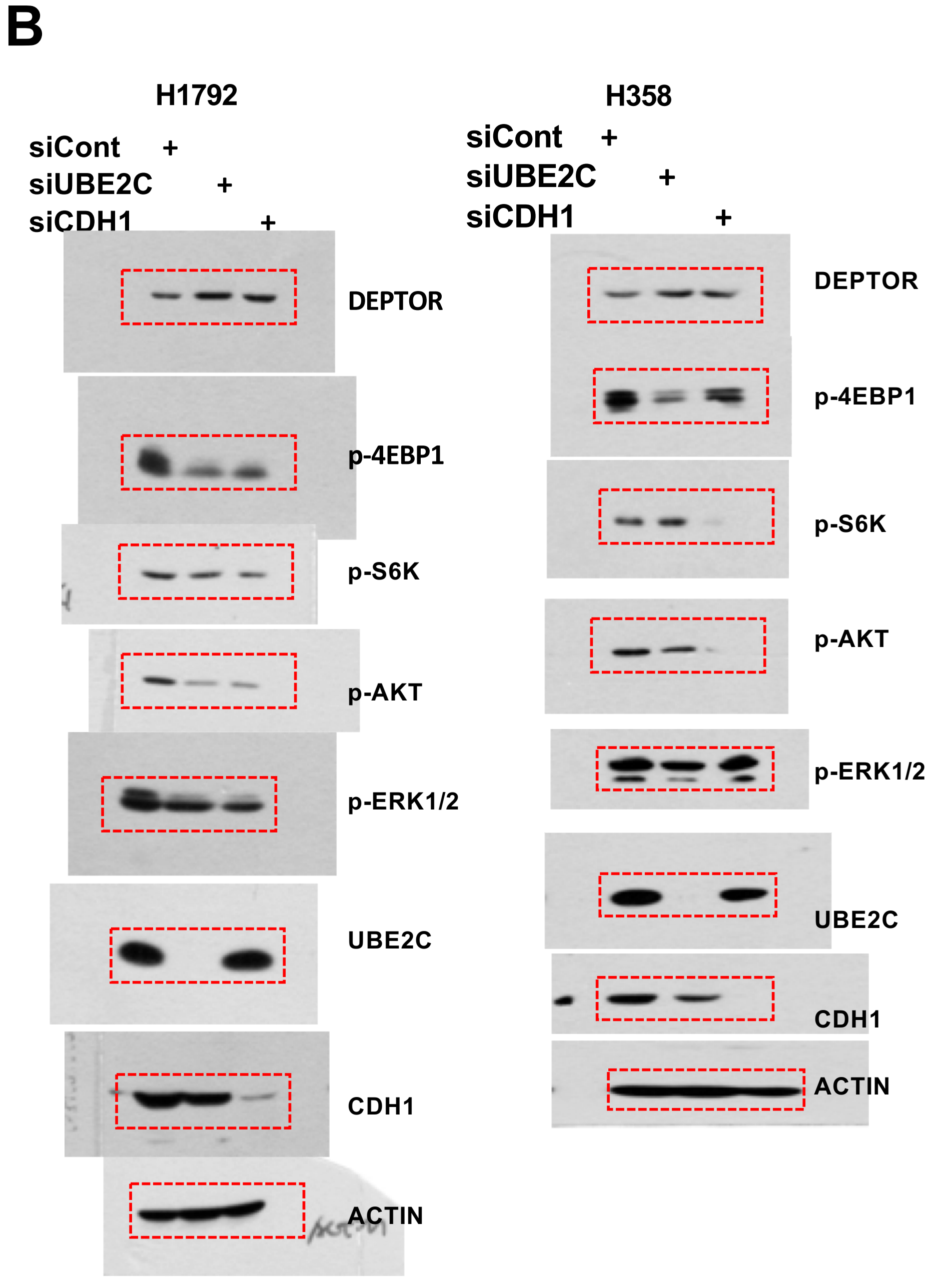

Full unedited gel for Figure S4

C

H1792

siCont siUBE2C

0 4 8 10 12 16 24 0 4 8 10 12 16 24h Thymidine released

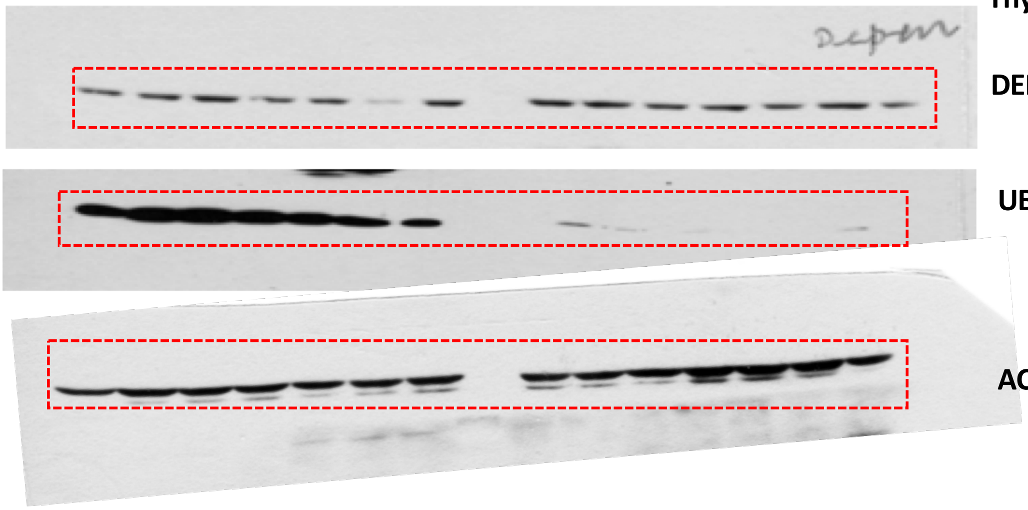

DEPTOR

UBE2C

ACTIN

D

H1792

siCont siCDH1

0 4 8 10 12 16 24 0 4 8 10 12 16 24h Thymidine released

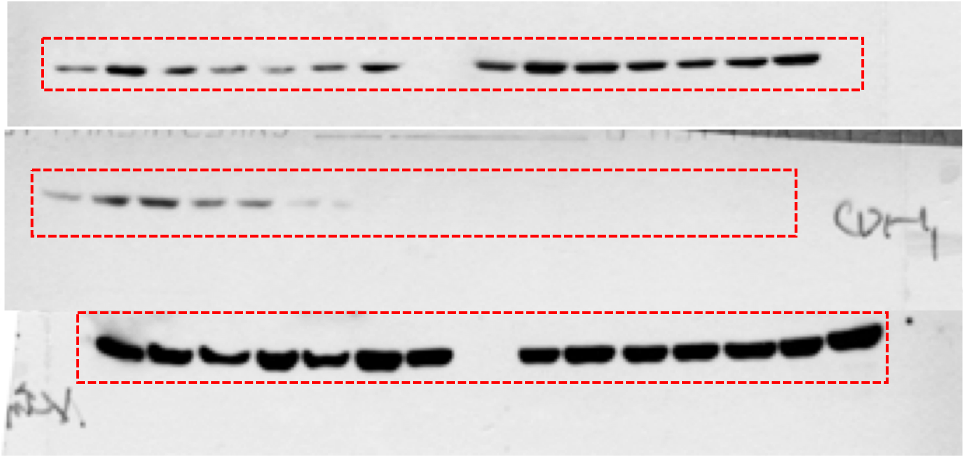

DEPTOR

CDH1

ACTIN

Full unedited gel for Figure S4

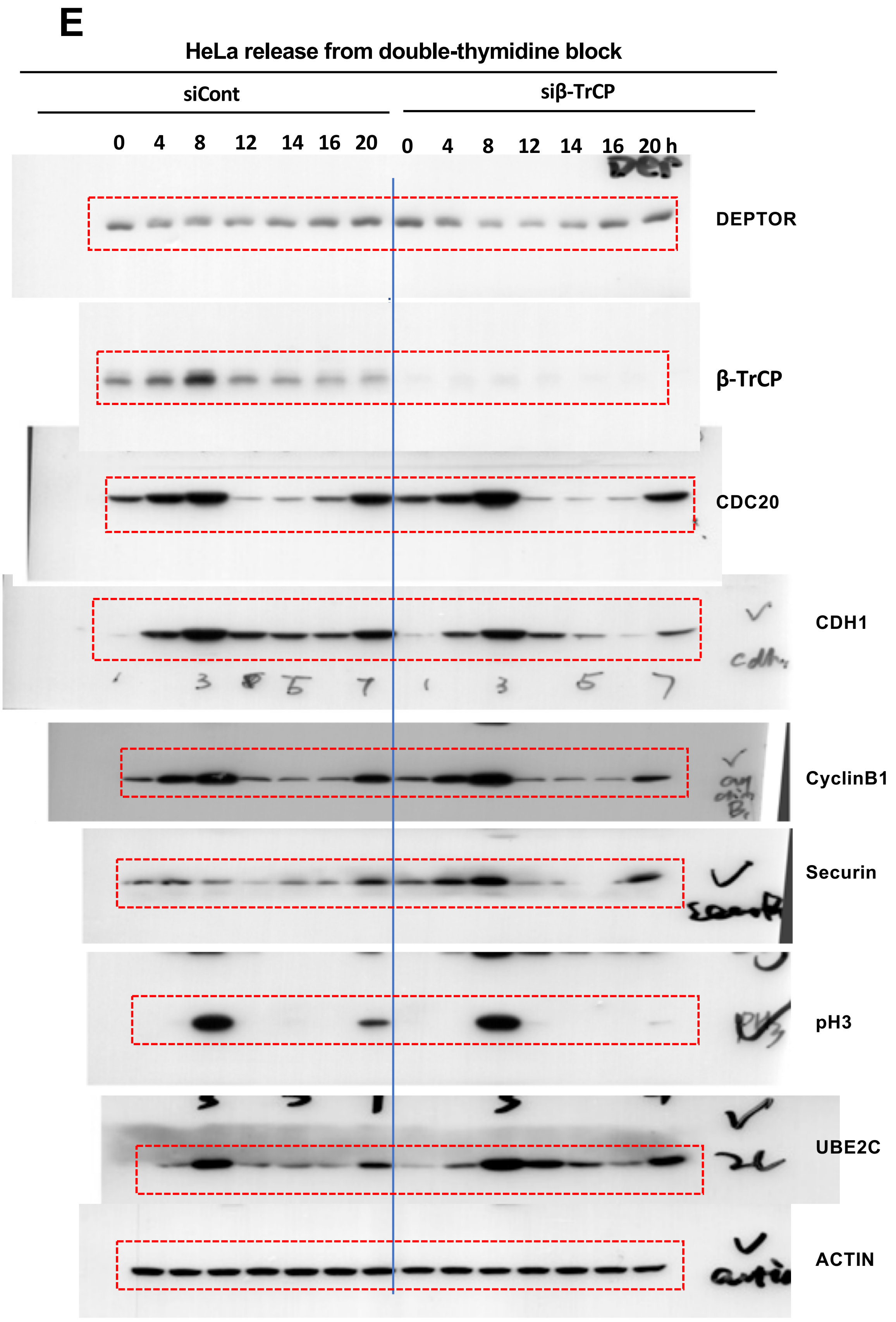

Full unedited gel for Figure S5

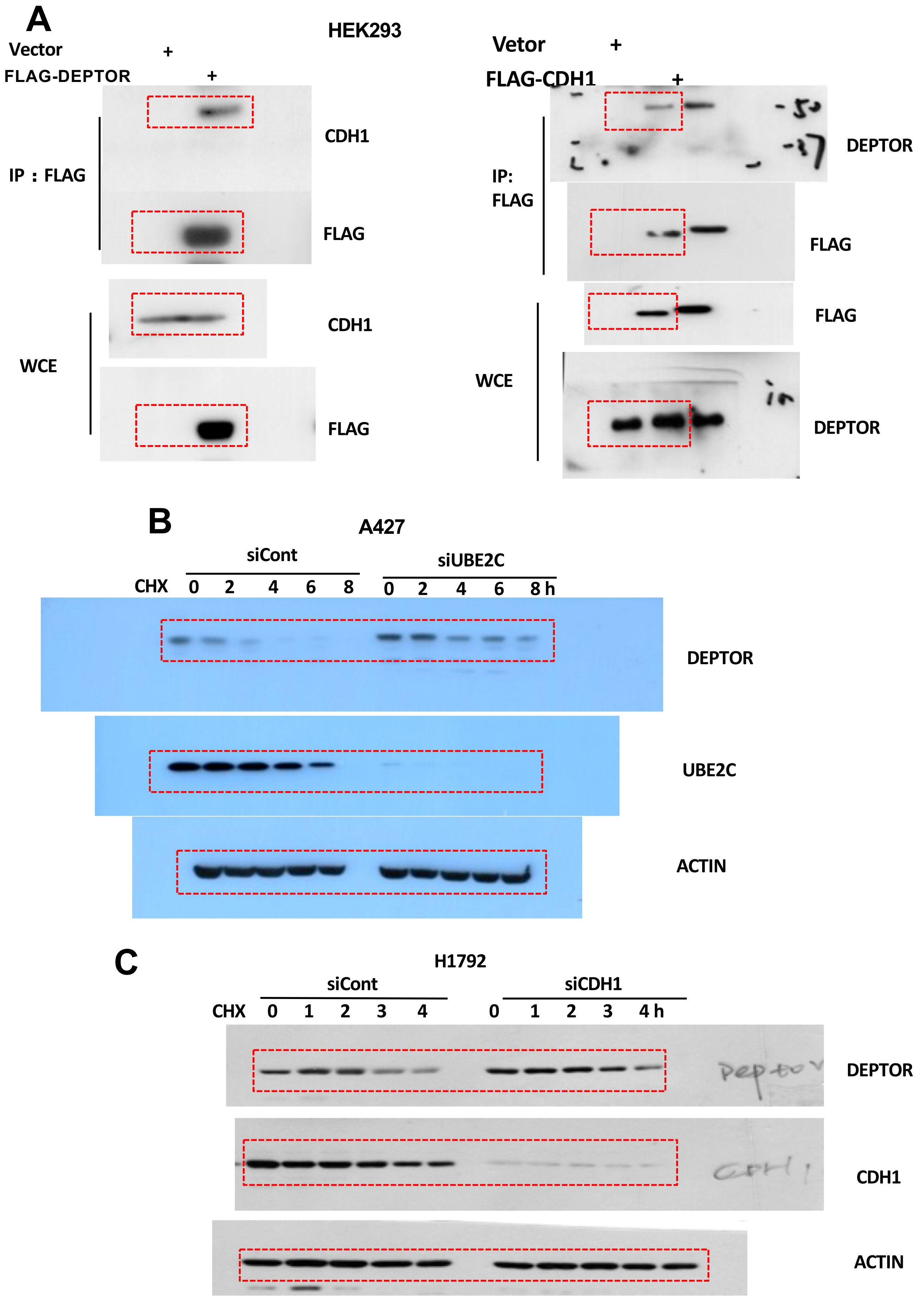

Full unedited gel for Figure S5

D

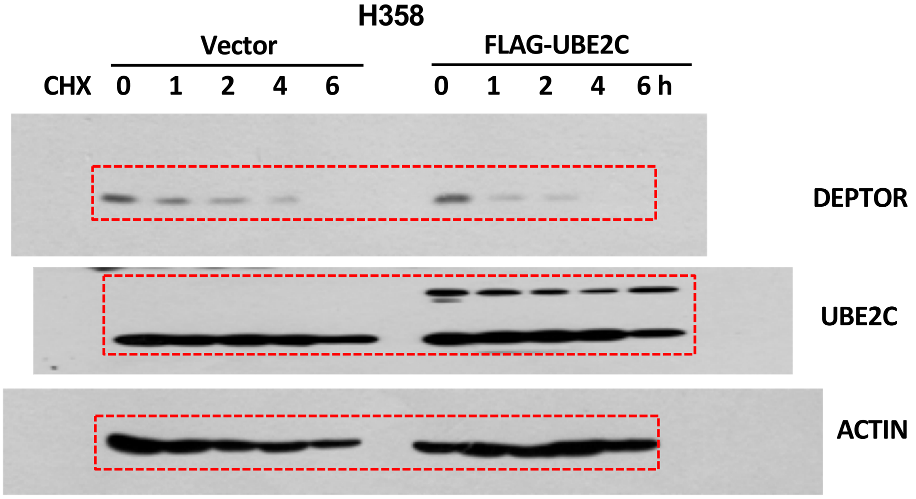

E

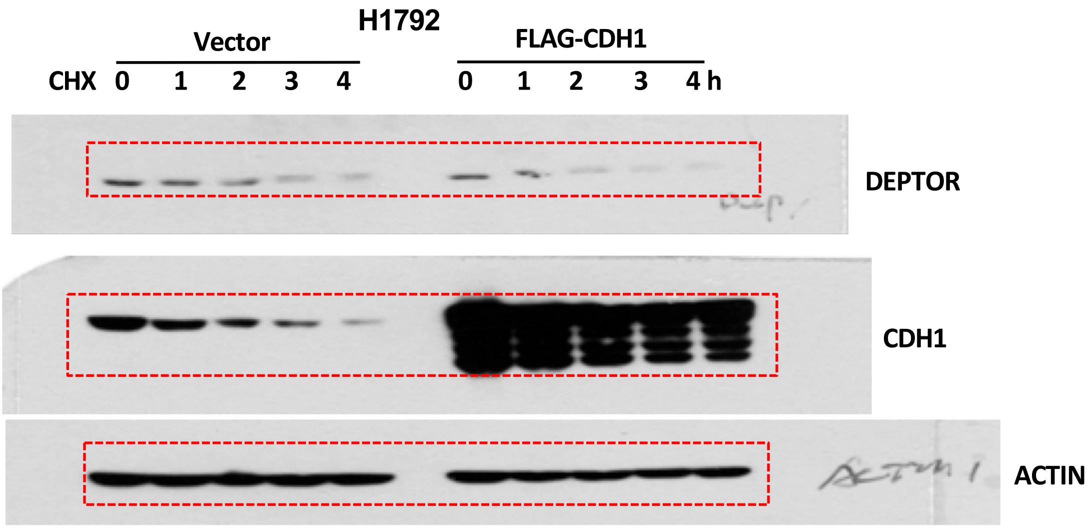

G

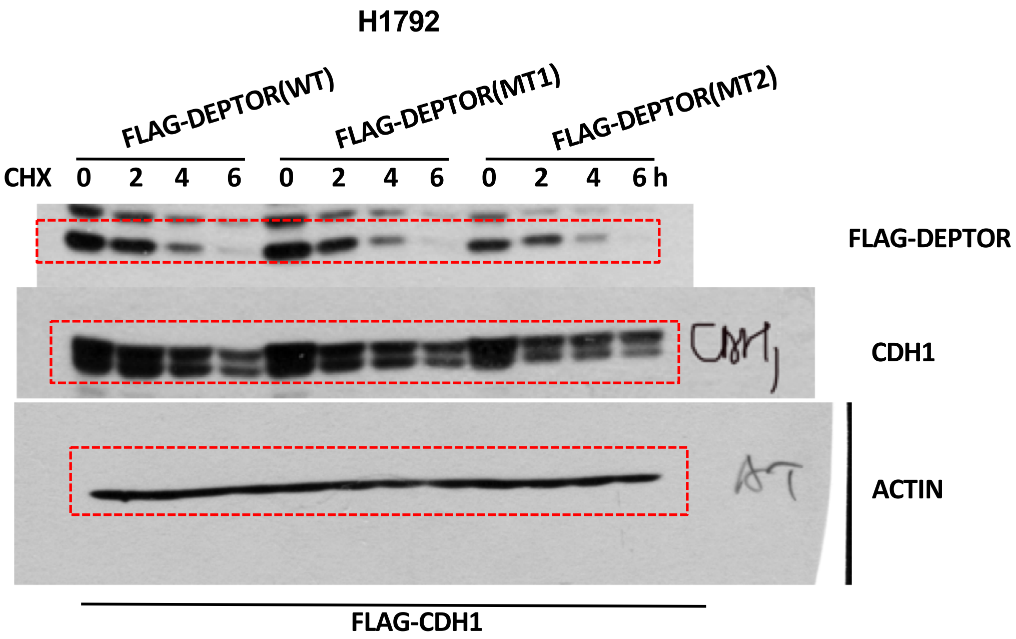

Full unedited gel for Figure S6

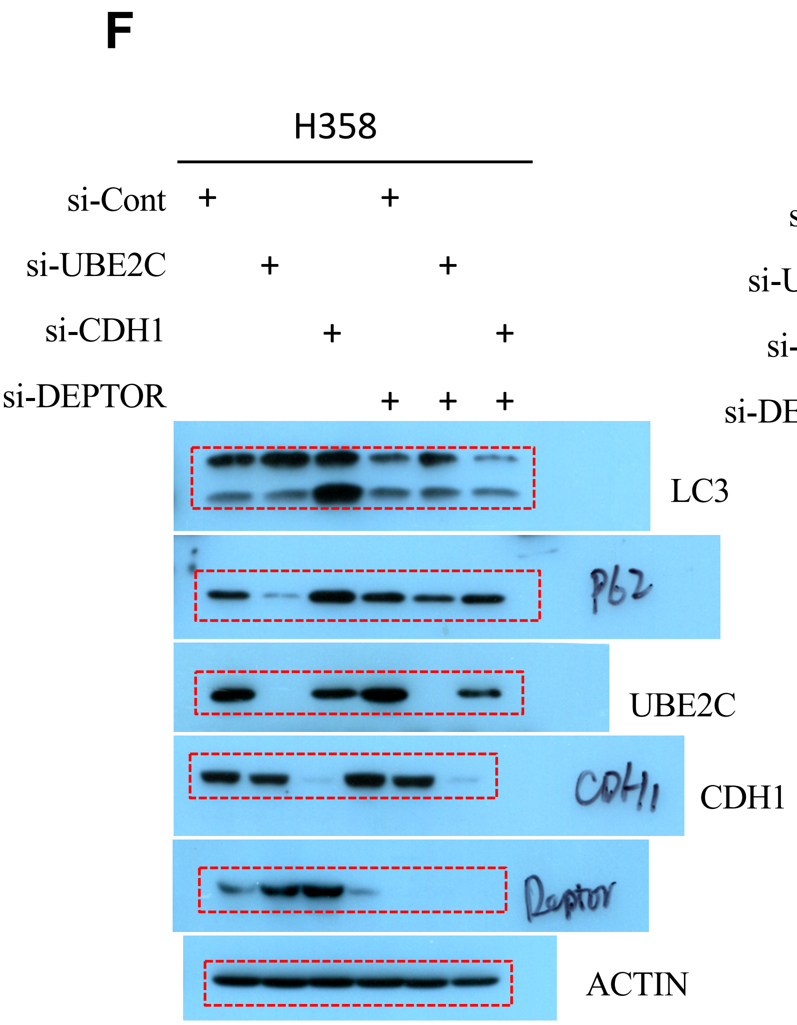

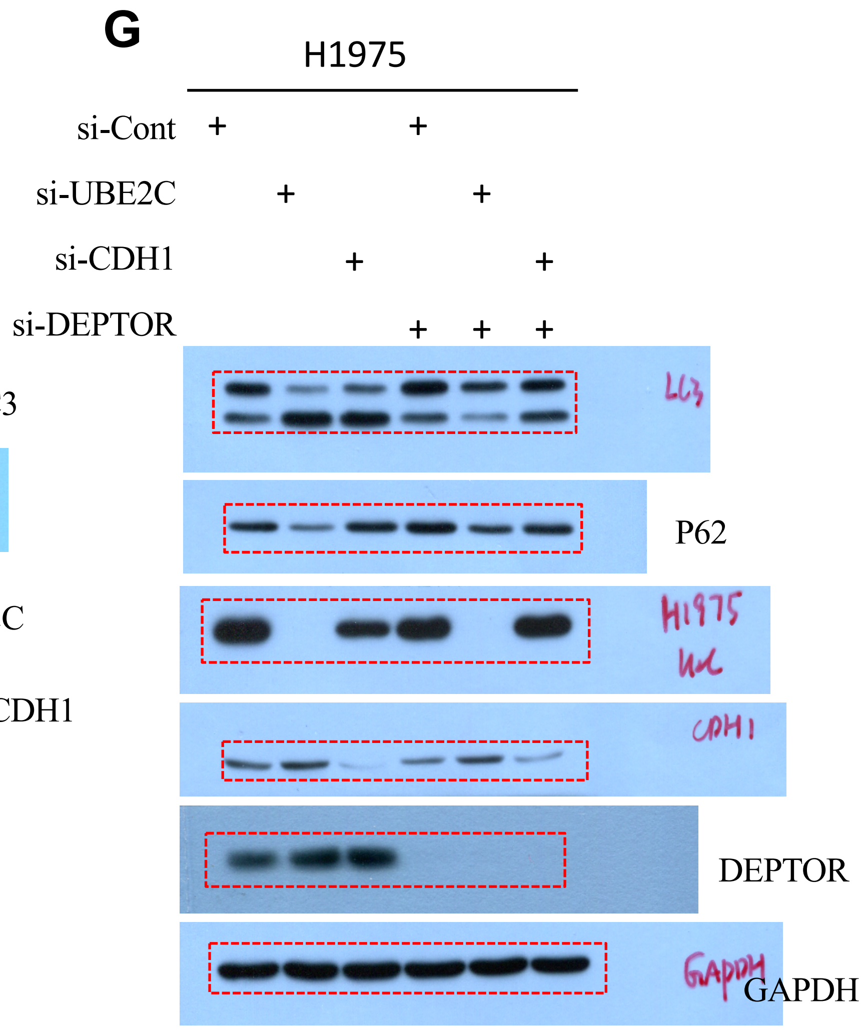

Full unedited gel for Figure S7

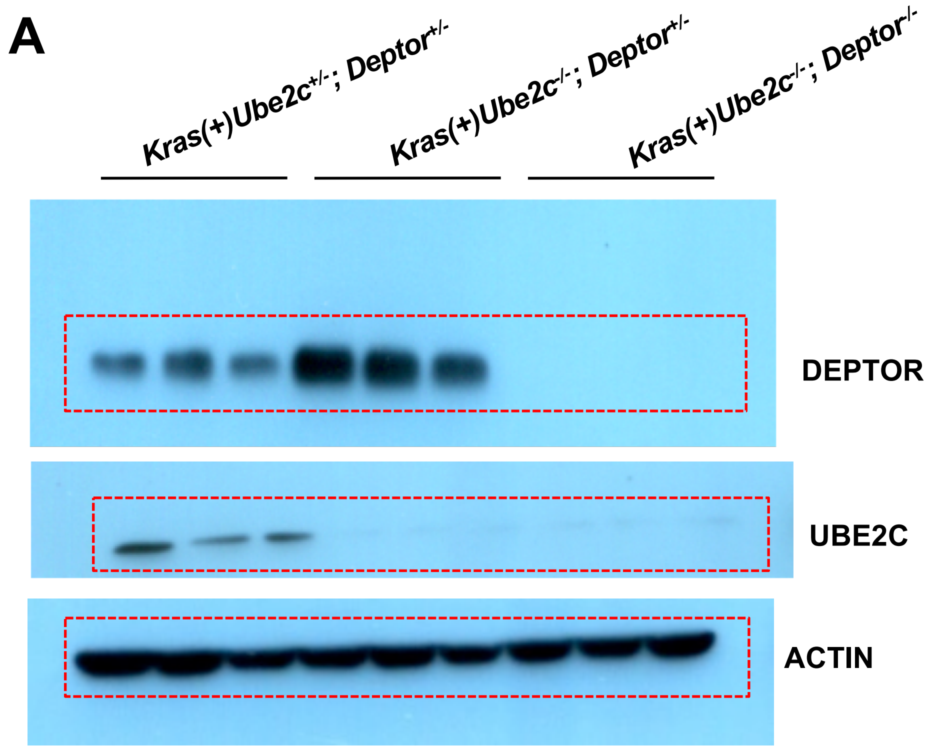

Supplement: Supplemental data [file jci-133-162434-s097.pdf]
